# Engineering osteoblastic metastases to delineate the adaptive response of androgen-deprived prostate cancer in the bone metastatic microenvironment

**DOI:** 10.1038/s41413-019-0049-8

**Published:** 2019-04-25

**Authors:** Nathalie Bock, Ali Shokoohmand, Thomas Kryza, Joan Röhl, Jonelle Meijer, Phong A. Tran, Colleen C. Nelson, Judith A. Clements, Dietmar W. Hutmacher

**Affiliations:** 1 School of Biomedical Sciences, Faculty of Health and Australian Prostate Cancer Research Centre (APCRC-Q), Institute of Health and Biomedical Innovation (IHBI), Queensland University of Technology (QUT), Brisbane, QLD 4000 Australia; 2 Translational Research Institute (TRI), Woolloongabba, QLD 4102 Australia; 3 Centre in Regenerative Medicine, QUT, Kelvin Grove, QLD 4059 Australia; 4 Bone and Joint Disorders Program, School of Chemistry, Physics and Mechanical Engineering, Science and Engineering Faculty (SEF), QUT, Brisbane, QLD 4000 Australia; 5 Australian Research Council (ARC) Training Centre in Additive Biomanufacturing, QUT, Kelvin Grove, QLD 4059 Australia

**Keywords:** Bone cancer, Bone cancer

## Abstract

While stromal interactions are essential in cancer adaptation to hormonal therapies, the effects of bone stroma and androgen deprivation on cancer progression in bone are poorly understood. Here, we tissue-engineered and validated an in vitro microtissue model of osteoblastic bone metastases, and used it to study the effects of androgen deprivation in this microenvironment. The model was established by culturing primary human osteoprogenitor cells on melt electrowritten polymer scaffolds, leading to a mineralized osteoblast-derived microtissue containing, in a 3D setting, viable osteoblastic cells, osteocytic cells, and appropriate expression of osteoblast/osteocyte-derived mRNA and proteins, and mineral content. Direct co-culture of androgen receptor-dependent/independent cell lines (LNCaP, C4-2B, and PC3) led cancer cells to display functional and molecular features as observed in vivo. Co-cultured cancer cells showed increased affinity to the microtissues, as a function of their bone metastatic potential. Co-cultures led to alkaline phosphatase and collagen-I upregulation and sclerostin downregulation, consistent with the clinical marker profile of osteoblastic bone metastases. LNCaP showed a significant adaptive response under androgen deprivation in the microtissues, with the notable appearance of neuroendocrine transdifferentiation features and increased expression of related markers (dopa decarboxylase, enolase 2). Androgen deprivation affected the biology of the metastatic microenvironment with stronger upregulation of androgen receptor, alkaline phosphatase, and dopa decarboxylase, as seen in the transition towards resistance. The unique microtissues engineered here represent a substantial asset to determine the involvement of the human bone microenvironment in prostate cancer progression and response to a therapeutic context in this microenvironment.

## Cancer: 3D model mimics prostate cancer metastasis to bone

A three-dimensional model of prostate tumor cells growing in a bone-like microenvironment offers a new platform for studying how metastatic prostate cancers respond to therapies. Dietmar Hutmacher and colleagues from the Queensland University of Technology in Brisbane, Australia, engineered a bone-like tissue environment by culturing bone progenitor cells in a fibrous polyester scaffold. The cells differentiated into bone-forming cells that produced the appropriate RNAs, proteins and minerals. The researchers then added various populations of prostate cancer cells to the constructs. Cell lines with the greatest bone metastatic potential grew best, and depriving cells of the hormone androgen led to a more aggressive disease phenotype consistent with that observed in the tumors of men with castration-resistant prostate cancer. The system offers a new way to test for relevant biomarkers and therapeutics in the laboratory.

## Introduction

Patients with metastatic castrate-resistant prostate cancer (CRPC) present with incurable bone metastases in 90% of cases.^1^ Bone metastases are the primary cause of morbidity and mortality in patients, with changes in the structural integrity of bone, associated with pain, debilitating skeletal-related events, and death.^2^ The interactions between bone microenvironment, rich in extracellular matrix proteins/stromal cells, and metastatic cancer cells, are an important component of the bone organ-specific progression of prostate cancer.^3^ Unfortunately, the current models lack the complexity of native bone tumor microenvironments, far from recapitulating individual aspects of the disease that enable the mechanistic advances needed to improve clinical outcomes.^4^

While rare prostate cancers, such as neuroendocrine, produce osteolytic or mixed osteolytic/osteoblastic lesions, metastases from most adenocarcinomas produce osteoblastic lesions, due to increased osteoblast activity.^5^ Prostate cancer influences bone homeostasis mostly by secreting paracrine factors that support osteoblast proliferation by direct effects (via growth factors—bone morphogenetic proteins, transforming growth factors, insulin growth factors),^3^ or by modifying factors present in the bone microenvironment (urokinase-type plasminogen activator, prostate-specific antigen (PSA)).^6^ PSA, the gold marker for prostate cancer progression, can indeed cleave various substrates in the bone microenvironment, including parathyroid-hormone-related protein,^7^ which in turn decreases bone resorption, making osteoblasts more predominant. While some growth factors expressed by osteoblasts are well-known to promote prostate tumor growth in the bone, a gap remains as many of these stimulating bone/tumor-derived factors have not yet been identified.^1^

With androgen signaling being key in prostate cancer, the use of androgen deprivation therapy (ADT) is the treatment of choice for patients with recurrent disease.^8^ While initially responsive, ADT ultimately leads to castrate resistance, by androgen receptor reactivation (AR) in cancer cells and adaption of stromal cells through direct sensitivity to AR targeting and paracrine interactions.^9^ With most patients remaining on androgen deprivation throughout their disease, ADT may contribute not only to cancer cell adaptation towards resistance, but may equally involve stroma adaptation favorable to metastasis progression. In the bone, androgen deprivation can indeed alter both osteoblastogenesis and osteoclastogenesis, negatively affecting the bone tumor microenvironment.^10^ Currently, defining the exact mechanisms behind cancer transition to castrate resistance in the bone microenvironment remains challenging, yet key to improving clinical outcomes.^11^ Considering the dominant role of osteoblasts in prostate cancer progression, a pre-clinical in vitro disease model that more closely mimics osteoblast/cancer interactions, is critical to investigate the adaptive response of this microenvironment in response to ADT.^4^

In the last two decades, the unmet need for better in vitro cancer models has drawn tissue-engineering technologies into the arena of cancer research.^12^ Tissue-engineered cancer models more faithfully recapitulate native three-dimensional (3D) microenvironments by better mimicking native structural and biochemical properties.^13^ Critical cell-to-cell and cell-to-matrix interactions can better be recreated using spatio-temporal approaches, ultimately leading to a more accurate study of a specific microenvironment. The ability to dissociate biological processes is equally important to gain insight into specific interactions between targeted cell populations. In the context of prostate cancer, where lesions are mostly osteoblast-driven, fundamental advances will be gained by separating the bone formation process from the bone resorption process, and opting for an osteoclast-free approach, as successfully justified previously.^14^

In this work, we present for the first time a tissue-engineered model that comprises both osteoblastic cells, osteocytic cells, and appropriate expression of osteoblast and osteocyte-derived proteins and mineral content. This model, viable long-term, can represent some of the key cellular and microenvironmental interactions between osteoblasts, their produces and prostate cancer for a more accurate study of osteoblastic bone metastases. The model is validated here by co-culture studies with metastatic prostate cancer cell lines, testing the hypothesis that the in vitro osteoblastic tumor microenvironment could reproduce some of the cellular alterations seen in vivo with androgen deprivation.

## Results

### Bioengineering of a human osteoblast-derived microtissue

**Fig. 1:**
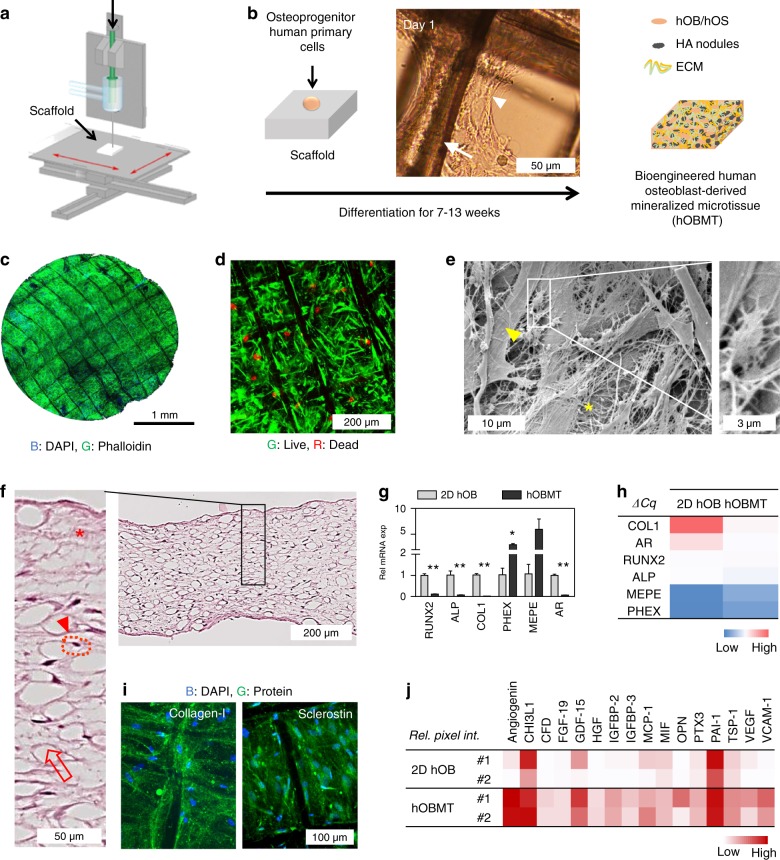
Bioengineering of a human osteoblast-derived microtissue and characterization. **a** Scaffold fabrication process using melt electrowriting and medical-grade polycaprolactone. Adapted with copyright from Farrugia et al.^15^ **b** Schematic of human primary osteoprogenitor cell seeding on a calcium phosphate-treated scaffold. The light micrograph shows scaffold fibers (arrow) and cell organization 1 day post seeding (arrow head). The culture of the cellular construct for at least 7 weeks leads to a human osteoblast-derived mineralized microtissue (hOBMT) containing live osteoblastic (hOB) and osteocytic cells (hOS), bone extracellular matrix (ECM) and hydroxyapatite (HA) mineralized nodules. **c** DAPI (blue) and Phalloidin (green) staining and confocal imaging (Max Proj image, 19 µm *z*-stack) of the hOBMT shows high cellular organization and strong directional actin filaments. **d** Live/dead FDA (green) and PI (red) staining and confocal imaging (Max Proj image, 100 µm *z*-stack) of hOBMT shows > 80% cell viability after 10 weeks in culture. **e** SEM imaging shows dense ECM deposition (asterisk), osteoblastic cells (arrow head), and osteocytic cells (inset). **f** Hematoxylin and eosin (H&E) staining on histology sections shows internal microtissue morphology, comprised of fibers (open arrows), osteocytic cells, surrounded by their lacunae (arrow head and dashed lines, respectively) and connective tissue (asterisk). **g** Gene expression of the hOBMT normalized to 2D cultures by RT-qPCR (*N* = 3, means ± SE) after 10 weeks of culture (*P < 0.05, **P < 0.01) with **h** heat map of ΔCq mean values. **i** Protein expression shows more and higher amounts of proteins (Angiogenin, thrombospondin 1 (TSP-1), vascular cell adhesion protein 1 (VCAM1), insulin-like growth factor-binding protein (IGFB-2)) expressed in the hOBMT compared to 2D hOBs (relative pixel intensity from protein microarray membranes) by heat map of means from two donors (#1, #2). **j** Confocal images of immunostained hOBMT for collagen-I (green) and sclerostin (green) and DAPI (blue), (Max Proj image, 50 µm *z*-stack)

Additive manufacturing and tissue-engineering technologies were combined to establish an in vitro osteoblast-derived microtissue model to study prostate cancer osteoblastic bone metastases. Scaffolds were 3D printed via melt electrowriting (Fig. 1a), and calcium phosphate coated (Fig. S1a) to achieve a morphology and surface chemistry favorable for cell culture and ECM/minerals deposition.^15^ Primary human osteoprogenitor cells were isolated from bone tissue and cultured on the scaffolds for up to 13 weeks (Fig. 1b), providing a cellular composite construct (Fig. 1c). The resulting human osteoblast-derived mineralized microtissue (hOBMT) displayed a typical human osteoblast (hOB)-type organization (Fig. S1b, c), with high viability (Fig. 1d) and dense extracellular matrix (ECM)/collagen-type fibrils deposition, with osteoblastic and osteocytic morphologies (Fig. 1e). Histological analysis showed a 3D tissue arrangement composed of connective tissue and homogeneous cellular distribution with cells surrounded by lacunae, as seen in vivo for osteocytic cells (Fig. 1f).^16^ At the messenger RNA (mRNA) level, reverse-transcription quantitative PCR (RT-qPCR) revealed the expression of a number of genes linked to osteoblastic differentiation and maturation [upregulation of alkaline phosphatase (*ALP*), parathyroid hormone 1 receptor (*PTH1R*), sclerostin (*SOST*), bone morphogenetic protein 1 (*BMP1*), osteoprotegerin (*OPG*), fibronectin (*FN*), *AR* and downregulation of osteonectin (*ON*) and collagen-I (*COL1*), Fig. S1d, e] in the differentiated hOBMT, and similar across donors (Fig. S1e). Comparison to mRNA levels with two-dimensional (2D) hOB revealed the mRNA signature of a more mature tissue observed in 3D (Fig. 1g, h).^17^

Particularly, the expression of central osteoblast genes (*RUNX2*, *ALP*) was reduced, while osteocytogenesis/osteocytic markers [phosphate regulating endopeptidase homolog X-linked (*PHEX*), matrix extracellular phosphoglycoprotein (*MEPE*)] were higher in hOBMT. Importantly, *AR* was downregulated (14-fold) in hOBMT. At the protein level, typical bone ECM (collagen-I), osteoblast mineralization (osteocalcin), as well as osteocyte (sclerostin) markers were expressed, as demonstrated by immunohistochemistry (IHC, Fig. S1f) and immunofluorescence (IF, Fig. 1i). Secretome analysis revealed that some proteins were only secreted in the hOBMT and/or in higher amounts than 2D hOB. This involved increased angiogenesis-related proteins [angiogenin, vascular endothelial growth factor (VEGF), thrombospondin (TSP-1)] and growth factors [fibroblast growth factor (FGF-19), hepatocyte growth factor (HGF), insulin-like growth factor-binding protein (IGFBP)-2, IGFBP-3, Fig. 1j].

Combined, this data illustrates the importance of using 3D platforms to obtain a more relevant and mature osteoblast-derived tissue microenvironment.

### Osteoblast-derived microtissues show increased maturation and mineralization over time

**Fig. 2:**
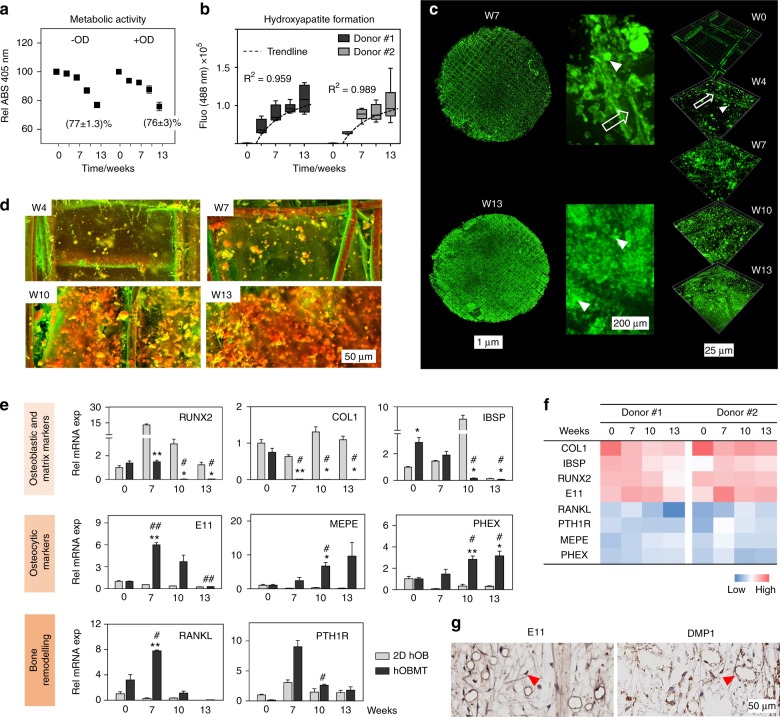
hOBMT mineralization and gene expression profile during osteoblastic maturation. **a** Metabolic activity of the hOBMT shows similar rates between undifferentiated microtissues (GM) and osteogenically differentiated microtissues (OM) over time (*N* = 3 donors, means ± SE). **b** Three-dimensional (3D) quantification of hydroxyapatite (HA) formation over time ($\bar N$ = 6, means ± SE), showing logarithmic correlation (*R*^2^ > 0.95). **c** Confocal images of hOBMT (donor #1) stained for hydroxyapatite (green), (Max Proj, 500 µm and 50 µm, *z*-stacks). Open arrows show fibers and arrow heads show deposited HA nodules. **d** Secondary electron and back scattered electron SEM images of hOBMT over time. The red/orange and green colors indicate denser and less dense material, respectively. Both **c** and **d** show increased mineralization over time. **e** Gene expression for 2D hOBs and the hOBMT over time, normalized to week 0 in 2D. Significance is shown for microtissues compared to 2D at each time point (*) and for the hOBMT at week 7, 10, and 13 compared to week 0 (^#^). *,^#^*P* < 0.05, **, ^##^*P* < 0.01. **f** Heat map of mean ΔCq values of hOBMT from two donors showing similar expression across genes. **g** Immunohistochemistry staining shows the expression of early (E11/podoplanin) and late [dentin matrix acidic phosphoprotein (DMP-1)] osteocytic cell markers in the hOBMT microtissues (arrow heads), after 7 weeks of osteogenic culture

Over 13 weeks of culture under osteogenic differentiation +/− (OD), the metabolic activity in hOBMT decreased, yet was above 75% (Fig. 2a), as expected when osteoblasts transition to osteocytes.^18^ Hydroxyapatite (HA) deposition occurred according to a logarithmic trend (Fig. 2b), with no statistical differences between 10 and 13 weeks osteogenic differentiation, and throughout the depth of hOBMT (Fig. 2c, d and Fig. S2a). The calcium to phosphorus (Ca:P) ratios of the microtissues were similar to that measured in the native bone from which the primary cells were isolated (Fig. S1b). No mineralization was observed on empty control CaP-coated scaffolds cultured in the same conditions (Fig. S2c), in line with osteoblast bio-mineralization, as seen previously,^19^ and as opposed to material-related physicochemical nucleation.

The analysis of mRNA levels over time showed that hOBMT reached osteoblast maturation and osteocyte differentiation earlier than 2D cultures, as seen by a decrease in osteoblastic, ECM and mineralization markers and increase in osteocytic and bone remodeling genes (Fig. 2e), with minor differences across donors (Fig. 2f). Compared to 2D, integrin-binding sialoprotein (*IBSP*) and *COL1*, secreted by maturing osteoblasts, decreased over time for hOBMT, as expected for mineralized matrices^20^ and during osteocytogenesis.^17^ The mRNA levels of osteocytic markers (*MEPE*, *PHEX*) increased significantly in hOBMT only, as expected when maturing osteoid osteocytes are present in the matrix.^21^ Bone remodeling genes (receptor activator of nuclear factor kappa-Β ligand (*RANKL*) and *PTH1R*) were maximum at week 7, before a gradual decrease concomitant with cellular entrapment and osteoblast-to-osteocyte transition.^22,23^ Early/intermediate osteocyte protein E11/Podoplanin, and intermediate/mature osteocyte protein DMP-1^24^ were expressed at both 7 and 10 weeks OD (Fig. 1g and Fig. S2c).

Altogether, hOBMT are viable long-term, highly mineralized, and able to partly display, contrary to 2D, the marker profile of osteocytogenesis.^21,23^

### Osteoblast-derived microtissues response to androgens and androgen deprivation

After 10 weeks osteogenic differentiation, the hOBMT were cultured in prostate cancer (PCa)-cell media for 3 weeks, to study the effects of androgen regulation on osteoblasts. PCa-cell media included PCa-Norm (environment prior to androgen deprivation), PCa-AD (no androgens) and PCa-DHT (physiological concentration of androgens, represented by dihydrotestosterone).

Without osteogenic supplements (Fig. S3a, b, c), proliferation, metabolic and ALP activity in hOBMT were lower in the supplement-depleted osteoblast growth medium (GM), which was similar to PCa-Norm. The mRNA levels of bone-specific genes (*COL1*, *ON*, *FN*), as well as *AR* and transforming growth factor beta1 receptor (*TGFB1R*), were overall unchanged without osteogenic supplements, in either 2D hOB and hOBMT (Fig. S3d). Exceptions were *ALP*, decreasing fivefold (both 2D hOB and hOBMT) and *SOST*, which increased eightfold (hOBMT only), in line with supplement removal, as *ALP* and *SOST* are positive and negative regulators of bone formation, respectively.^16^ Increase in *SOST* was observed in hOBMT only because of the osteocytic cell profile higher than 2D, hence promoting expression when osteogenic supplements were removed.^21^

**Fig. 3:**
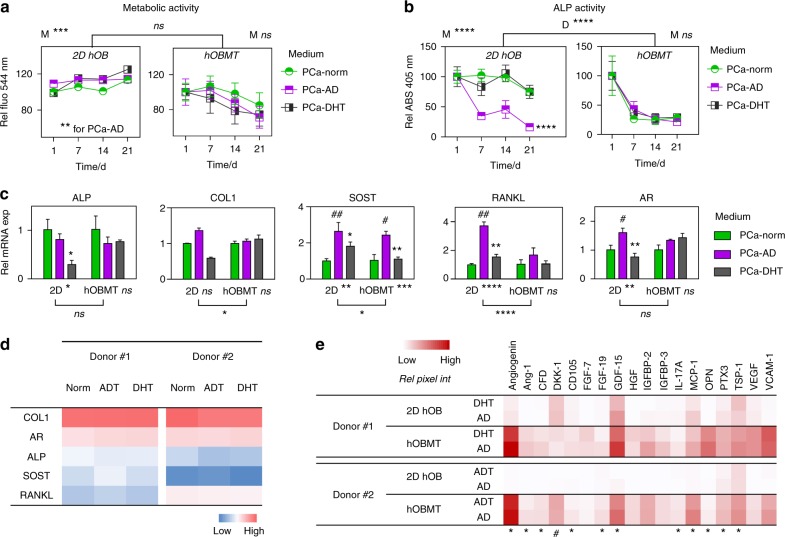
hOBMT function, mRNA, and protein levels in the presence of dihydrotestosterone (DHT) and in androgen-deprived conditions. **a** Metabolic activity and **b** alkaline phosphatase (ALP) activity in medium for the hOBMT and 2D hOBs after 10 weeks of osteogenic maturation, followed by 21 days culture in prostate cancer (PCa) media. Expressed as % to Day 1 control (PCa-Norm). Day 1 refers to the first day following the 10 weeks of normal hOBMT maturation ($\bar N$ = 4, means ± SE). *P-*values are compared to PCa-Norm. *M* shows the *P-*values for overall effect of Medium. **c** Gene expression for 2D hOBs and the hOBMT after 10 weeks of osteogenic maturation, followed by a 10-day treatment in PCa media. Fold changes are normalized to PCa-Norm for each dimension. Hashtags (^#^) show significance for PCa-AD compared to PCa-Norm, and asterisks (*) show significance for PCa-DHT compared PCa-AD (*N* = 3, means ± SE). **d** Heat map of mean ΔCq values for two donors, showing similar gene expression variations across media. Blue = low gene expression, red = high gene expression. **e** Protein expression shows similar expression from PCa-AD to PCa-DHT media within each dimension (2D and hOBMT), as measured by relative pixel intensity from microarray membranes shown as heat map of means from two donors (#1, #2). The asterisks and hashtags refer to <20% increase or >20% decrease from DHT to AD medium, respectively

Metabolic activity was similar across all media conditions (Fig. 3a), although hOBMT presented with reduced trend over time. As seen in Fig. 2a, this is due to continuous osteoblast-to-osteocyte transition, where osteocytes are less metabolically active, compared to osteoblasts.^21^ ALP activity in the 2D setting decreased in the PCa-AD group only (****, Fig. 3b). In hOBMT, a similar drop was observed (****), but this was similar across all media conditions. Again, this decrease is expected as ALP is a by-product of osteoblastic activity, highly expressed in pre-osteoblasts and osteoblasts but not expressed by transitional cells and osteocytes.^18^ At the mRNA level (Fig. 3c), overall differences between 2D hOB and hOBMT were observed for *COL1*, *SOST* (*), and *RANKL* (****), with none observed for *ALP* and *AR*. In PCa-AD, *RANKL*, and *AR* were lightly upregulated in 2D (3.7-fold, 1.6-fold) although this was not observed for hOBMT, highlighting the differences between 2D and 3D settings. *SOST* was the only gene upregulated in 2D and 3D in PCa-AD ( > 2.4-fold), in line with direct *AR*-mediated effect, with androgen deprivation leading to *SOST* increase.^25^ Overall, PCa-DHT led to similar response as in PCa-Norm in hOBMT, and similar between donors (Fig. 3d).

At the protein level (Fig. 3e), androgen deprivation led to a slight increase in angiogenesis-related cytokines (angiogenin, VEGF, endoglin (CD105), TSP-1), growth factor expression (FGF-19 and growth/differentiation factor-15 (GDF-15)) and bone protein osteopontin (OPN).

Overall, the data demonstrates that the hOBMT platform allows for long-term co-culture experiments (up to 28 days) in PCa cell-based media in contrast to 2D co-culture experiments, which fail on many levels after 3–4 days.

### Co-culture with PCa cells show morphometric and functional differences under androgen deprivation

**Fig. 4:**
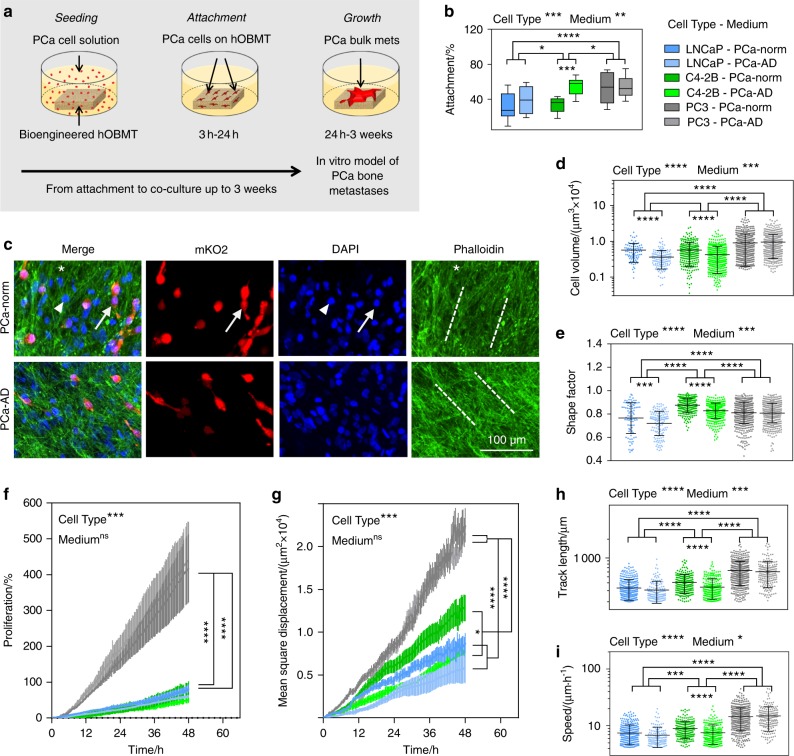
hOBMT co-culture with human prostate cancer (PCa) cell lines show quantitative morphological and functional differences according to cell type and androgen presence. **a** Schematic of the co-culture of hOBMT with PCa cells, showing how PCa cancer cell are seeded overnight, attaching during the first 24 h. Once attached, cells keep proliferating to form micro-aggregates at the surface of the hOBMT, up to 3 weeks post seeding. **b** Attachment rates of LNCaP, C4-2B, and PC3 cells conditioned for 7 days in PCa-Norm and PCa-AD, after 24 h of co-culture in the respective media ($\bar N$ = 10, box-and-whisker plots). **c** Confocal images of metastatic microtissues after 24 h of co-culture, showing mKO2-LNCaP cells aligning to the hOBMT ECM in both PCa-Norm and PCa-AD medium (Max Proj, 70 µm *z*-stack). Split channels show LNCaP cells (mKO2, red), mixed hOBs and LNCaP nuclei (DAPI, blue) and actin filaments (Phalloidin, green), and merged images. Solid arrows show LNCaP cells, arrow heads show hOBs, asterisks show the hOBMT and dashed lines show the direction of ECM deposition. **d, e** Morphometric properties of the cancer cells on hOBMT after 24 h co-culture, shown as scatter plots; **d** PCa cell volume, **e** PCa cell shape factor (*N* = 3, with each *N* comprising three random fields of view with > 40 cells per field of view, i.e., $\bar n$=335 cells per condition. Means ± SD). Shape factor values range from 1 (round) to 0 (fully elongated). **f** PCa proliferation and **g–i** migratory properties on the hOBMT over 48 h post attachment; **g** Mean square displacement; **h** Track length, **i** Cell speed (*N* = 3, 8–10 fields of view, $\bar n$ = 270 cells tracked per condition. Means ± SE). Over significance is shown for Cell type and Medium. Stars (*) show local significance between the groups and sub-groups

The hOBMT were used in co-culture with AR-positive and dependent (LNCaP), AR-positive and independent bone metastatic (C4-2B), and AR-negative bone metastatic (PC3) cell lines, in androgen-replete and androgen-deprived contexts (Fig. 4a). To validate the osteoblast-derived model, the hypothesis was that androgen deprivation would affect AR-positive cells (LNCaP/C4-2B) but be ineffective on AR-negative PC3 cells.

After seeding on hOBMT, pre-conditioned metastatic cell lines were homogeneously distributed across all cell lines/conditions (Fig. S4a). Attachment rates (Fig. 4b) revealed that PC3 attached highest and were unaffected by medium (53% ± 5% and 54% ± 3%), while LNCaP and C4-2B attached at lower rates, with more attachment for C4-2B (*). Androgen deprivation led to increased LNCaP and C4-2B attachment rates (32% ± 5% vs. 39% ± 5% for LNCaP, 34% ± 2% vs. 54% ± 3% for C4-2B), although significantly only for C4-2B (***). This suggested that androgen deprivation predisposed AR + LNCaP cells to attach better to bone tissue in the initial stage of metastasis. C4-2B under androgen deprivation also attached at similar rates as PC3, suggesting the acquisition of androgen-independent features upon androgen deprivation. In all groups, within 24 h, cancer cells attached and spread along the ECM fibrils at the surface of hOBMT (Fig. 4c and Fig. S4b), with no differences in orientation on hOBMT (Fig. S5a).

The morphometric features of cancer cells can inform on cell plasticity and malignancy^26^ and be used to evaluate adaptive phenotype.^27^ In the context of prostate cancer, it is well-established that prostate cancer cells escape androgen deprivation by processes such as epithelial-to-mesenchymal transition (EMT) or neuroendocrine transdifferentiation (NEtd),^28–30^ using pseudopodial actin dynamics,^27^ with highly deformed spindle-like phenotypes. Such distinct morphological features (increased cellular volume, decreased shape factor) are hallmarks of transition to resistance and hence represent a useful tool for quantification. Here, we established a methodology to assess the morphometric parameters of LNCaP, C4-2B, and PC3 after 24 h of co-culture with hOBMT in normal and androgen-deprived conditions. We hypothesized that the largest phenotypic change would be seen with the most androgen-responsive cell line (LNCaP), a moderate response observed with C4-2B and no/little effects on PC3 cells, as being AR negative.

The results disclosed that PC3 had the highest cellular volume (Fig. 4d and Fig. S5b, c), and were indeed not affected by androgen deprivation. The volumes of both LNCaP and C4-2B cells were smaller than PC3, and equivalent. Yet, reduced volume and decreased shape factor (Fig. 4e) were observed in PCa-AD medium. LNCaP had the smallest shape factor across all cell lines with the smallest value under androgen deprivation (0.72 ± 0.01), showing that the highest degree of AR responsiveness led to the most spindle-like phenotype in response to androgen deprivation in the bone microenvironment, proving our hypothesis.^29,30^

Cancer cell migration and proliferation are important components of metastasis progression. Here, live cell imaging was exploited to track prostate cancer cells on hOBMT for 48 h. PC3 cells had the highest proliferation rates in PCa-Norm (418% ± 96%) compared to LNCaP and C4-2B cells, which were similar (84% ± 11% and 80% ± 22%, respectively, Fig. 4f). Although reduced in trend, no statistical differences in proliferation were observed for any cell line under androgen-deprived conditions (Fig. S5c). Next, we analyzed mean square displacement (MSD), cellular speed (SP), track length (TL), and track straightness ratio (TS) as key migratory properties (Fig. 4f–i and S5b, c, Video S1). TS was similar across all conditions and cell types, showing similar track directionality on hOBMT (Fig. S5d). PC3 migrated the most [(22.5 ± 2.0) × 10^3^ µm^2^, (14.5 ± 0.3) μm·h^–1^ and (627 ± 12) µm, respectively for PCa-Norm] and were unaffected by androgen deprivation conditions (Fig. 4g–i and Fig. S5c). C4-2B and LNCaP had intermediate and low migratory properties, respectively. Remarkably, while C4-2B were affected by androgen deprivation, there was no significant impact of androgen deprivation on LNCaP. For instance MSD was (8.8 ± 1.1) × 10^3^ µm^2^ for LNCaP-norm and (7.7 ± 1.0) × 10^3^ µm^2^ for LNCaP-AD, so that although migration was slower, androgen deprivation did not statistically affect the migration of LNCaP cells on hOBMT. On the other hand, the more aggressive C4-2B cells migrated more [(12.8 ± 1.5) × 10^3^ µm^2^] in the normal media, but with androgen deprivation, reduced migration [(7.4 ± 1.0) × 10^3^ µm^2^ distance traveled, ****] was observed to a similar level seen for LNCaP cells [(7.7 ± 1.0) × 10^3^ µm^2^].

Here, we proved the hypothesis that only the AR + cell lines studied (LNCaP and C4-2B) were affected by androgen deprivation in the osteoblast-derived microenvironment. While no overall significant effect was observed for LNCaP’s dynamic properties, C4-2B were affected by androgen deprivation. Since LNCaP would be affected by androgen deprivation in a mono-culture setting, these results clearly highlight the possible contribution of the osteoblast-derived microenvironment to androgen-responsive LNCaP survival.

### Lncap show the highest adaptive response in osteoblast-derived microtissues

**Fig. 5:**
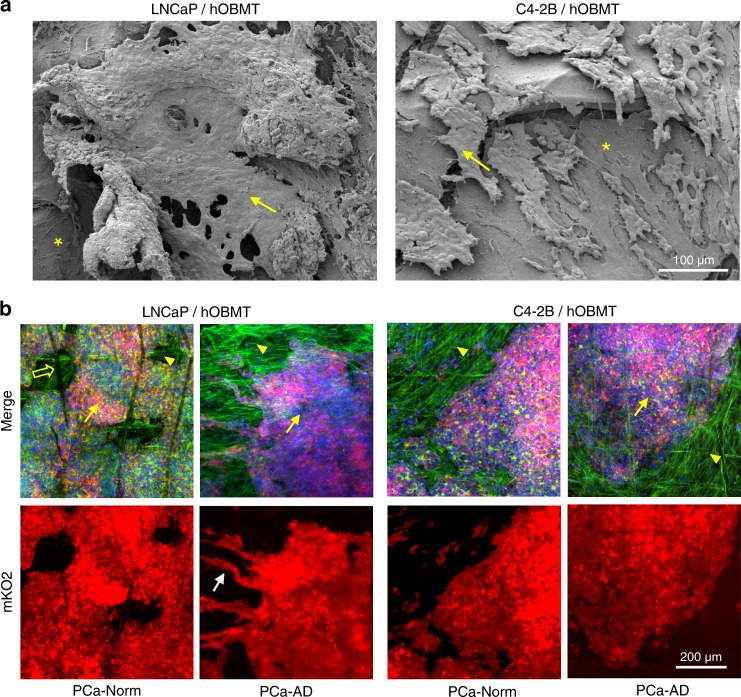
Morphologies of LNCaP and C4-2B cells after long-term co-culture with the hOBMT. **a** SEM images show that LNCaP and C4-2B cells attached and aligned onto the surface of the hOBMT, adhering to each other and to the hOBMT to form bulk micro-metastatic aggregates by 21 days of culture. The asterisks show the hOBMT and the arrows show the cancer cells. **b** Confocal images show the corresponding 3D morphologies (Max Proj, 70 µm *z*-stack) of aggregated cancer cells. C4-2B cells display similar morphologies between PCa-Norm and PCa-AD medium, but LNCaP aggregates show sprouting of cancer cells from bulk (white arrow) in PCa-AD. Split channels show LNCaP and C4-2B cells (mKO2, red), and merged images (mKO2 in red, DAPI in blue, Phalloidin in green). The open arrow shows a scaffold fiber, the arrow heads show the hOBMT and the full arrows show the cancer cells

Over long-term co-culture, single cancer cells aggregated within the first week of co-culture (Fig. S6a, b and Fig. S7a), forming prostate cancer aggregates on the surface of the hOBMT. By 21 days of co-culture, ~70% of the hOBMT was covered by LNCaP or C4-2B in PCa-Norm (Fig. 5a and Fig. S6a). C4-2B aggregates did not display morphological differences under androgen deprivation when compared to PCa-Norm (Fig. 5b and Fig. S6c), and remained compact. Conversely, LNCaP aggregates branched out from the bulk onto hOBMT fibrils and displayed highly elongated morphologies typical of EMT and/or NEtD^28–30^ at 21 and 28 days of co-culture (Fig S7b). These cells displayed the longest cellular protrusions (>75 µm) and a high degree of alignment with the underlying hOBMT.

To our knowledge, we are the first to establish a model that allows to study the adaptive features of androgen-responsive LNCaP cells under androgen deprivation in an osteoblast-derived microenvironment.

### Co-culture with cancer cells dysregulates bone tumor microenvironment markers

RNA and proteins from co-cultures were collected and compared to monocultures, after culture in DHT + / −. Dose response was determined by *AR* and *PSA* gene expression analysis (Fig. S8a) and selected as 10 nmol·L^–1^ DHT.

**Fig. 6:**
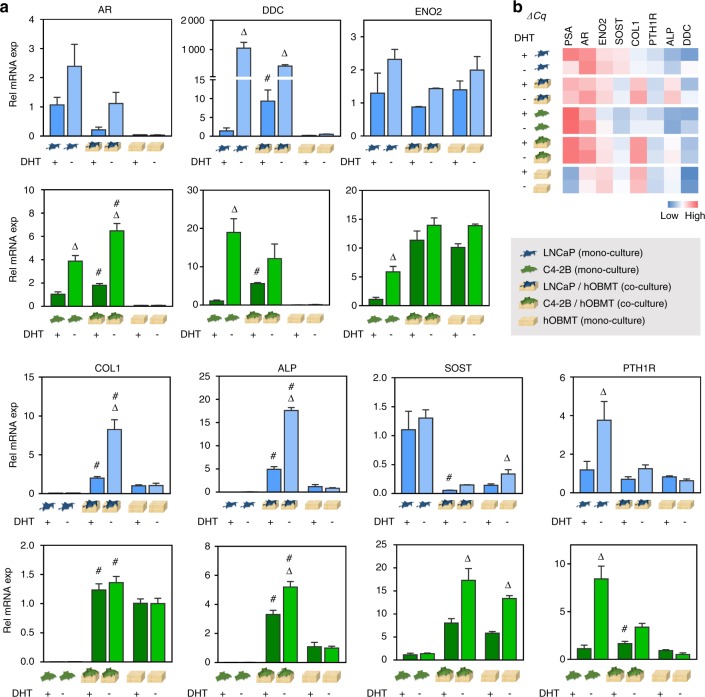
Gene regulation of prostate cancer cell (PCa)/hOBMT co-cultures. **a** Relative mRNA expression levels of PCa cell monocultures, hOBMT and PCa/hOBMT co-cultures (LNCaP dataset in blue, C4-2B dataset in green), in PCa-DHT (10 nM DHT) and PCa-AD (no DHT). Gene fold is normalized to PCa cell monocultures in PCa-DHT for all genes, except collagen-I (*COL1*) and alkaline phosphatase (*ALP*) (normalized to hOBMT in PCa-DHT). *N* = 3, means ± SE. Delta signs (^Δ^) show significance from PCa-DHT to PCa-AD within each sub-group. Hashtags (^#^) show significance of PCa/hOBMT co-cultures to both PCa cell monocultures and hOBMT, for each medium condition. **b** Corresponding heat map of mean ΔCq values

Gene analysis (Fig. 6) revealed mRNA changes in *AR*, bone-relevant genes (*COL1*, *ALP*, *SOST*, *PTH1R*) and NEtD-relevant genes (dopa decarboxylase (*DDC*) and enolase 2 (*ENO2*)). In the androgen setting (‘‘DHT + ’’), the ECM/mineralization markers *COL1* and *ALP* displayed significant upregulation in both co-cultures compared to hOBMT, in line with the clinical situation.^31^ *SOST* was significantly downregulated in the LNCaP/hOBMT co-cultures compared to LNCaP or hOBMT alone, in line with more prostate cancer progression in the bone.^32^ *PTH1R* was up-regulated in C4-2B/hOBMT co-cultures, but constant in LNCaP/hOBMT. *DDC*, a neuroendocrine marker of prostate cancer linked to progression to castrate resistance^33^ was also upregulated in both co-cultures. *ENO2* had a similar trend in C4-2B/hOBMT cultures at the gene and protein levels (Fig. 6 and Fig. S9).

Many changes were observed under androgen deprivation (‘‘DHT−’’). While *PSA* was significantly lower (Figs. S8a and S9) in prostate cancer cells and co-cultures, genes associated with progression to aggressive disease and resistance to therapy^9,29^ were upregulated in both co-culture types: *AR*, *DDC*, and *ENO2*. Conversely, epithelial-to-mesenchymal transition-relevant genes (*Slug*, *Snail*, *Zeb-1*) were found unaltered under androgen deprivation in the co-cultures (not shown). *AR*, known to amplify during androgen deprivation to facilitate tumor cell growth in low androgen concentrations^33,34^ was expressed significantly higher in all settings at both gene and protein levels. *PTH1R*, a key regulator in tumor–bone interactions, was upregulated under androgen deprivation in prostate cancer mono-culture and co-cultures. *PSA* was highly downregulated by androgen deprivation, as expected (Fig. S8a), but LNCaP/hOBMT co-cultures were still 3.3-fold over-expressed, compared to LNCaP alone (Fig. S8b), demonstrating the contribution of the bone microenvironment in maintaining *PSA* expression.^35^ The strongest effects of androgen deprivation were for *ALP* and *COL1*, where androgen deprivation had no effect on hOBMT alone, but had a striking effect on co-cultures, with LNCaP/hOBMT most affected. This is in line with increased ALP serum levels in patients under androgen deprivation therapy.^36^

**Fig. 7:**
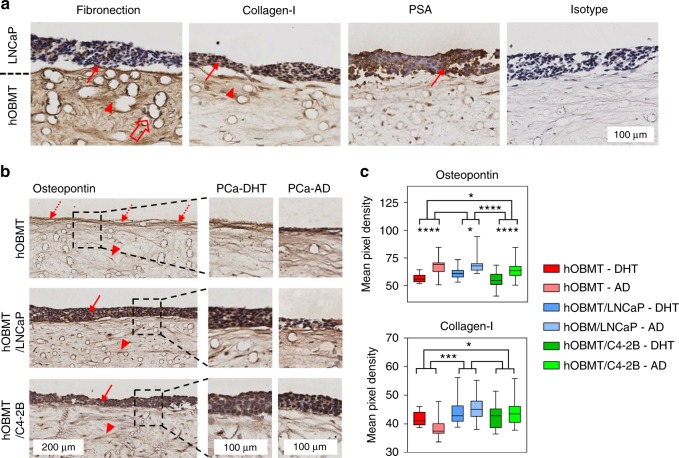
Protein expression in prostate cancer cell (PCa)/hOBMT microtissues. **a** Immunohistochemistry shows the expression of fibronectin, collagen-I, and prostate-specific antigen (PSA, brown), and counterstained nuclei (purple) in LNCaP/hOBMT co-cultures, after 10 days of co-culture in PCa-Norm medium. Solid and head arrows show staining in the LNCaP bulk and the hOBMT, respectively. While PSA is found in the LNCaP cells only, Fibronectin is found mostly in the hOBMT. Collagen-I is found in both the LNCaP cells and the hOBMT with increased staining in hOBMT close to the LNCaP cells. **b** Immunohistochemistry images of the hOBMT and PCa/hOBMT co-cultures, stained for osteopontin show increased staining towards the surface, for the hOBMT (dashed arrows), and staining in both PCa cell bulk (solid arrows) and the hOBMT (head arrows). Less metastatic burden is observed in LNCaP/hOBMT in PCa-AD, while it is unchanged for C4-2B/hOBMT. **c** Quantification of mean pixel densities (non-zero values) in the hOBMT areas only (scanning of the hOBMT alone and the hOBMT area only in co-culture with LNCaP and C4-2B cells), showing increased staining in the hOBMT in all conditions under AD for osteopontin. Collagen-I is also more expressed in the hOBMT when co-cultured with either LNCaP or C4-2B ($\bar N$= 10 sections/condition, with three fields of view/section for full section coverage, means ± SE displayed (*P < 0.05, **P < 0.01, ***P < 0.001, ****P < 0.000 1)

Finally, we used quantitative IHC to confirm protein expression levels (Fig. 7). While PSA was strongly found in LNCaP (Fig. 7a), fibronectin was found mostly in hOBMT. Collagen-I was found in both LNCaP and hOBMT with increased staining in hOBMT close to the LNCaP areas. Analysis of the staining intensity in hOBMT (Fig. 7b, c) revealed that osteopontin, a known contributor of prostate cancer progression,^37^ was over-expressed by androgen deprivation in both hOBMT alone (previously confirmed by protein array analysis) and co-cultures. As seen at the mRNA level, collagen-I was over-expressed in both co-cultures compared to hOBMT alone, with increased staining under androgen deprivation (Fig. 7c), validating our findings from the gene to the protein level.

## Discussion

In the clinical management of advanced prostate cancer, radiological, and histological evidence show that all adenocarcinoma prostate cancer types that have metastasized to the bone present with osteoblastic/sclerotic lesions, found in osteoblast-rich areas in the form of woven bone.^3,10^ This finding has allowed to co-treat skeletal-related events with radiopharmaceuticals, such as radium-223 chloride, by inhibiting bone metastases through preferential deposition at sites of increased osteoblast activity, in turn increasing patient survival rates.^1^ Conversely, the clinical co-targeting of osteoclasts, responsible for a fraction of osteolytic metastases, has indicated that osteoclasts only had a secondary role in prostate cancer progression. Clinical trials with agents such as zolenodrate or denosumab, which inhibit osteoclast activity, showed that osteoclast-targeting agents could reverse bone loss from hormonal therapies, yet did not slow down the progression of bone metastases,^3^ demonstrating that osteoblasts remain one of the key drivers in prostate cancer bone metastasis.^38^

Androgen deprivation therapy is inevitable for patients with recurrent disease, and is maintained throughout disease progression, despite inducing resistance at the metastatic sites^38^ and higher mortality.^39^ The mechanisms behind this adaptation involve the reactivation of AR-related pathways and cancer/stroma interactions.^40^ Consequently, Phase III clinical trials for metastatic CRPC comprise typical androgen blockade strategies, chemotherapy and bone-homing radiopharmaceuticals or growth factor signaling pathway [Platelet-derived growth factor (PDGF), FGF, HGF] inhibitors.^3^ Although briefly prolonging patient survival, these therapies ultimately only provide palliative benefits. Importantly, at the early stage of androgen deprivation therapy, whether or not prostate cancer has progressed to bone yet, the systemic suppression of androgens has severe implications for the bone organ, with loss of mineral density, high remodeling rates, and a higher risk of fractures.^1^ What is currently unknown is whether further endocrine alterations as a side-effect from hormonal therapies may contribute to progression to castrate resistance in this microenvironment. Emphasis needs to be given to models that can reproduce androgen deprivation and demonstrate similarities to the clinical scenario, where bone metastases are found in patients already under ADT.^40^ This undertaking will be crucial to identify additional factors that will improve the clinical situation.^1^

Three-dimensional (3D) in vitro models of bone metastases have become increasingly recognized to enhance the current knowledge, which is built on 2D culture models and in vivo animal models of skeletal metastasis.^41^ The former lack the complex interactions between cancer cells and the physicochemical bone microenvironment, which, in vivo, play a critical role in directing and maintaining cell fate,^42^ and the latter do not reproduce the biological programs specific to human disease.^41^ Ultimately, both models have limited potential to delineate novel contributors or be used efficiently to predict clinical outcome.^4^ While the paradigm is shifting towards using more relevant in vitro 3D models, few attempts have been made, to date, to apply these models to answer relevant biological questions.^14^ In particular in the bone microenvironment, the balance between bone formation and bone resorption makes it challenging to engineer a system that enables to identify the individual contributions of each cell type towards tumor survival and progression. As seen previously,^14^ it is mechanistically more relevant to initially dissociate the bone formation/resorption processes, before increasing complexity by considering them simultaneously. Specifically in the context of prostate cancer bone metastasis, which is mostly driven by osteoblasts, many responsible factors have still not been identified^3^ and hence an approach that is osteoclast-free has a stronger rationale.^4^

Here, we have used tissue-engineering strategies to propose a novel in vitro 3D model of direct osteoblasts/prostate cancer cells interactions, which can be used quantitatively to address biological hypotheses related to osteoblastic bone metastases. One strength of this model is rooted in its capability to perform long-term studies and osteoblast-derived microtissues displaying the morphological features of a highly mineralized mature tissue. The presence of osteocytic cells enabled the expression of mature bone markers, which is often not seen in 2D, and not well-designed 3D, models.^21^ Osteocytes are multifunctional cells with many regulatory roles in bone and mineral homeostasis^43^ and have recently been implicated in prostate cancer bone metastases.^4,44^ Osteocytes influence bone remodeling in both osteoblastic and osteolytic lesions, although little is known about their roles in osteotropic cancer bone metastasis.^4^ The glycoprotein sclerostin, which we identified at the gene/protein levels here, is only expressed by mature osteocytes.^21^ Sclerostin plays an important role for the catabolic activity of bone by controlling the *RANK*/*OPG* mRNA ratio, which ultimately affects prostate cancer bone metastases.^45^ Here, the ability of our platform to provide long-term cell viability, as well as high-mineral deposition in a 3D setting was essential to trigger osteoblast-to-osteocyte transition by osteoblast burial within the matrix,^18,21^ and led to successful osteocytogenesis, as seen at the mRNA and protein levels.

Direct-contact 3D cancer assays are critical to assess cell-to-cell and cell-to-matrix interactions. Biophysical, as much as biochemical, interactions indeed promote cancer transition towards resistance, with explicit adaptive phenotype.^27^ In processes controlled by pseudopodial actin dynamics,^27^ such as epithelial-to-mesenchymal transition and neuroendocrine transdifferentiation, cancer cells display distinct morphological features of the adaptive response, which ultimately informs about transition to castrate resistance.^28^ Using quantitative measures, such as cell volume, shape factor or orientation on matrix, it is hence possible to identify, within the microenvironment, what conditions lead to such phenotypes.^26,27^ With the set of quantitative methodologies established here, we demonstrated that PC3, the most prolific cell line used in the osteoblast-derived microtissues, did not respond to androgen deprivation, and that LNCaP and the castrate-resistant sub-line C4-2B behaved more similarly and how they responded to androgen deprivation. LNCaP presented clear adaptation with the appearance of NEtD features, which was correlated with the increased expression of NEtD markers, *DDC*, and *ENO2*. As an AR co-activator, *DDC* enhances AR activity and plays a central role in castrate resistance progression.^33^ NEtD is a known adaptive pathway that contributes to the development of CRPC,^46^ and it was demonstrated here for the first time in a 3D in vitro model of osteoblastic bone metastases. Importantly, *DDC* upregulation was already occurring in androgen-replete conditions, as a result of co-culture, validating the bone contribution in initiating adaptive response mechanisms prior to androgen deprivation.

We further presented evidence that the osteoblast-derived microenvironment was supportive of the AR-positive and dependent LNCaP cells by reducing only slightly cancer cell proliferation and migration, but not significantly, as would have been expected at the start of ADT.^29^ This may be explained by the lower migration rate of LNCaP cells, compared to more aggressive C4-2B. The cells still reached similar proliferative, migratory, and morphometric properties as C4-2B under androgen deprivation. Higher migratory properties seen for C4-2B in androgen-replete conditions corroborated the fact that C4-2B are past the transition to castrate resistance and derived from bone metastases formed by LNCaP, hence are used to growing in this microenvironment. Importantly, a vast majority of castration-resistant cases are not truly depleted of androgens and still use androgens to maintain cell proliferation and tumor growth,^38^ hence the effects observed here on C4-2B.

The osteoblast-derived microenvironment showed active participation to the adaptive transition of cancer cells. Under androgen deprivation, *PSA* was upregulated in LNCaP/hOBMT co-cultures, a direct consequence of osteoblast-secreted factors, such as interleukin-6, that cause androgen-independent induction of *PSA* gene expression. This process contributes to maintaining LNCaP proliferation and migration in the bone microenvironment by a mechanism that partially relies on AR.^35^ Co-culture of AR responsive LNCaP and C4-2B cells with hOBMT under androgen-replete conditions generated over-expression of important bone markers found in clinical samples (COL1) and serum levels (ALP), the latter a common feature of patients with bone metastatic prostate cancer.^47^ *SOST* downregulation, concomitant to *ALP*/*COL1* upregulation, is in line with the profile of osteoblastic bone metastases seen in advanced prostate cancer.^38^ After androgen removal, these changes (*ALP*, *COL1*, *SOST*) were more accentuated, but mostly in LNCaP/hOBMT co-cultures, due to LNCaP’s higher degree of AR responsiveness. As *SOST* is expressed by some prostate cancer cells, circulating sclerostin levels are usually significantly increased in prostate cancer patients and particularly in those receiving ADT,^48^ as androgens are key regulators of bone metabolism in this population. Consistent with these findings, *SOST* expression, provided here by the osteocytic population from hOBMT was heightened by androgen deprivation,^25,48^ also explaining the reduced migration observed for C4-2B under androgen deprivation. In fact, *SOST* has an inhibitory effect on prostate cancer invasion,^32^ which induced lower rates of metastasis,^49^ and a lack of *SOST* within bone promotes expression of genes associated with cell migration/invasion.^32^ Finally, even when *SOST* expression was heightened by androgen deprivation, overall expression levels from co-cultures were kept low (C4-2B/hOBMT) or strongly downregulated (LNCaP/hOBMT), compared to monocultures. This is the demonstration of a clear adaptive response from the bone tumor microenvironment to aid metastasis progression. In future, it will also be important to investigate other metastatic cell lines, such as VCaP, DuCaP, or C4-2, to further unravel the effects of androgen deprivation for those metastatic variants in the osteoblast-derived microenvironment. Finally, the use of patient-derived tissues (xenografts, prostatectomy samples) in co-culture with the microtissues would be warranted in the future as a predictive platform for the testing of current and novel therapeutics for individual patients.

Overall, the tissue-engineered model combined with the quantitative methodologies presented here form the basis of an authoritative novel pre-clinical platform to interrogate osteoblasts/cancer cells interactions. Technical challenges remain, as direct-contact models provide a complex milieu, which challenges downstream analyses,^42^ with difficulties including the recovery of mRNA and proteins. Importantly, the proposed model focused on prostate cancer interactions with osteoblasts, osteocytes, and their respective ECM, due to the pathological relevance of osteoblastic lesions, yet some prostate cancer types present with mixed osteoblastic and osteolytic lesions.^50^ Osteoclasts indeed rely on *RANKL*, *OPG*, and matrix metalloproteinases, among others,^51^ which may all be expressed by prostate cancer cells, ultimately affecting the crosstalk with osteoblasts, in turn influencing the resulting predominant lesion type. Hence it will be valuable to have a complementary model that includes osteoclasts in the future.^52,53^ Bone cells/cancer cells interactions via direct-contact is however often critical to achieve system relevance, yet this causes characterizations issues when identifying contributing cell populations, so a system that enables to test both contributions individually and simultaneously may be ideal.^51^

In conclusion, although every in vitro model is imperfect by definition,^14^ this study represents a significant advancement in the field, as it addresses some of the key challenges in engineering osteoblast-derived metastatic microenvironments.^4^ These improvements include the capacity for patient specificity, the presence of a mature mineralized tissue with osteoblastic and osteocytic cells, with clinically relevant gene and protein expression, and the quantification of prostate cancer cell morphology and function in this microenvironment. The validation of the model’s responsiveness to androgens and androgen deprivation, with observations akin to the clinical scenario, warrants a more accurate study of the transition to castrate resistance and implications in the osteoblastic tumor microenvironment. Ultimately, this will lead to more rapid discovery and relevant testing of novel biomarkers and drugs for the treatment and/or prevention of osteoblastic prostate cancer.

## Materials and methods

### Bioengineering of a human osteoblast-derived microtissue

The manufacturing of medical-grade polycaprolactone (mPCL) microfiber scaffolds (10 × 10 mm, 600 µm thickness, 12 µm fiber diameter, 150 µm pore size) was performed via melt electrowriting (MEW), using a custom in-house built apparatus (IHBI, QUT, Brisbane, Australia), as per established protocols.^54^ Printed scaffolds were coated with calcium phosphate (CaP). Isolation of human osteoprogenitor cells from donor bone tissue was in accordance with QUT ethics approval number 1400001024. Osteogenic potential was validated by alizarin red staining (Fig. S10). Isolated cells were seeded at passage 4–5 on sterilized scaffolds (0.4 × 10^6^ cells/scaffold) and differentiated osteogenically for 10 weeks, unless otherwise specified. The bioengineered constructs are referred to as human osteoblast-derived microtissues (hOBMT). In parallel, for 2D controls, 3 000 cells/cm^2^ were seeded in 6-well plates, and treated similarly to hOBMT. Two-dimensional (2D) osteoblast cultures are referred to as ‘2D hOB’. For specific details, see the Methods Supplement.

### Osteoblast-derived microtissue characterization

The surface morphology of the hOBMT was obtained by scanning electron microscopy (SEM). 3D mineralization was quantified using an OsteoImage mineralization assay. 3D morphology and expression of bone protein markers were determined by immunofluorescence (IF) and immunohistochemistry (IHC). 3D fluorescence imaging was done using spinning disc confocal microscopy (SDC). Physicochemical characterization was investigated using energy dispersive X-ray spectroscopy (EDS) and SEM in secondary-/back scattered-electron mode. Osteoblast viability, DNA content, metabolic activity and alkaline phosphatase (ALP) expression in medium were measured using a Live/Dead staining assay with fluorescein diacetate (FDA) and propidium iodide (PI), PicoGreen dsDNA quantification assay, PrestoBlue cell viability assay, and SigmaFAST kit, respectively. See the Methods Supplement.

### Cancer cell lines

Human prostate cancer cell lines, LNCaP, C4-2B, and PC3, were sourced from ATCC. Cells from passages 18–35 were used. Routine culture was in RPMI-1640 medium + l-glutamine, no phenol red, containing 5% fetal bovine serum (FBS) and 1% penicillin/streptomycin (P/S), all from Gibco. For fluorescence analysis, cells were transduced with a pLEX307-mKO2 plasmid (kindly donated by Dr. Sally Stephenson, QUT), and positive cells were selected using puromycin (1 µg·mL^–1^). All cells were cultured in a humidified incubator (37 °C, 95% air, 5% CO_2_).

### Androgen treatments and co-culture conditions

Normal medium used for co-culture experiments contained RPMI, 10% FBS, and 1% P/S. This medium is referred to as ‘PCa-Norm.’ To mimic androgen deprivation (labeled as ‘AD’), FBS [containing 0.6 nmol·L^–1^ dihydrotestosterone (Sigma-Aldrich), (DHT)] was replaced with 10% charcoal stripped serum (CSS), from Gibco (containing undetectable traces of DHT, as per manufacturer’s certificate of analysis). This medium is referred to as ‘PCa-AD.’ ‘PCa-DHT’ medium refers to the PCa-AD medium supplemented with 10 nmol·L^–1^ DHT.

### Co-culture with prostate cancer cells

Prostate cancer cell suspensions were prepared as 1 × 10^5^ cells/mL for LNCaP and C4-2B cells, and 0.5 × 10^5^ cells/mL PC3 cells, in PCa-Norm media. The hOBMT were placed in 24-well plates and supplemented with 500 µL of cell suspensions. After 24 h of co-culture on a rocking platform mixer (RPM4, Ratek Laboratory Equipment), supernatants were aspirated and microtissues washed, before analysis. A seeding variant was performed to quantify prostate cancer cell attachment to hOBMT (See the Methods Supplement).

### Morphometric analysis

Co-culture microtissues were fixed in 4% paraformaldehyde (PFA, Sigma-Aldrich) for 40 min, after 24 h of co-culture in PCa-Norm or PCa-AD media, and stained for DAPI and phalloidin. SDC was used to image the cancer cells (mKO2, red), the nuclei (DAPI, blue), and the F-actin filaments (phalloidin, green). The 10 × Plan Apo objective was used, with the red (ex 561 nm), green (ex 488 nm), and blue (ex 405 nm) filter sets. Maximal intensity projections were made from *z*-stacks using 1 µm as step size and 70 µm thickness (>2 microtissues/condition analyzed with > 3 fields of view, for ~335 cells analyzed/condition). Cancer cell volume and shape factor were obtained from Imaris imaging analysis software (version 9.1.0, Bitplane AG, Zurich, Switzerland) and cancer cell orientation on hOBMT was obtained from ImageJ software (1.51j8. NIH, USA,^55^). Algorithm details are found in the Methods Supplement.

### Live-cell maging and analysis

After attachment, the co-culture microtissues were placed in a new 24-well-plate and secured down using Teflon ring inserts (Prestige Manufacturing Pty Ltd). A live-cell inverted epifluorescence microscope (IX81, Olympus) fitted with a humidified chamber, 95% air, 5% CO_2_, set at 37 °C, was used to collect images every 20 min for 48 h. Fluorescent signal from prostate cancer cells was used to track movement on the hOBMT. Migration analysis was performed using Imaris and proliferation analysis with ImageJ (>2 microtissues/condition analyzed with ~8 fields of view/microtissue, for ~ 270 tracks analyzed/condition). Algorithm details are found in the Methods Supplement.

### Gene analysis

RT-qPCR was first used to quantify gene expression differences between 2D hOB and hOBMT over time. Second, RT-qPCR was done on 10-week-old hOBMT cultured for an extra 10 days in osteogenic, PCa-Norm, PCa-AD, and PCa-DHT media. Third, RT-qPCR was performed on the PCa/hOBMT after 10 days co-culture with either LNCaP or C4-2B in either PCa-AD or PCa-DHT media and compared with monocultures, cultured in the same conditions. At collection point, RNA was collected and extracted, reverse transcribed, and processed for RT-qPCR as detailed in the Methods Supplement. The list of primers is found in Table 1. Expression of target mRNA was determined using the delta-delta Cq method, using the geometric average of *7SL* and *RPL32* reference genes. Results are expressed as means ± standard error (SE) from three independent experiments/donor.

**Table 1:** Primer sequences used for RT-qPCR

Target gene (HUGO nomenclature)	Primers (5′-3′)	Fragment size/bp
*RUNX2*	F: CCTCCTACCTGAGCCAGATG R: ATGAAATGCTTGGGAACTGC	165
*ALP* (*ALPL*)	F: ACCATTCCCACGTCTTCACATTTG R: AGACATTCTCTCGTTCACCGCC	162
*COL1* (*COL1A1*)	F: AGGGACACAGAGGTTTCAGT R: AGCACCATCATTTCCACGAG	188
*PHEX*	F: TATCTTGAAGCTGGACCAAGCA R: ACTTGTAAAGGGCATCCCGA	101
*MEPE*	F: GGGCCTGCCCATTCCTTCTCGT R: ACCCCAGGAGCCTTTCCCTTGTG	178
*PSA* (KLK3)	F: AGTGCGAGAAGCATTCCCAAC R: CCAGCAAGATCACGCTTTTGTT	138
*AR*	F: CTGGACACGACAACAACCAG R: CAGATCAGGGGCGAAGTAGA	245
*BMP1*	F: CTTCTGGCACGAACACACTCG R: ACTCCTGCCCTGGCTGGAT	80
*ON* (*SPARC*)	F: CAAATACATCCCCCCTTGCC R: GATCTTCTTCACCCGCAGCTT	151
*OPG* (*TNFRSF11B*)	F: TTCCGGAAACAGTGAATCAA R: CGCTGTTTTCACAGAGGTCA	287
*FN* (*FN1*)	F: CAGTGGGAGACCTCGAGAAG R: TCCCTCGGAACATCAGAAAC	168
*PTH1R*	F: GCGGAGCTGAGGAGACGTAG R: CTTCCCTGATGTGGACGCAG	185
*SOST*	F: AGAGTACCCCGAGCCTCC R: AGCTGTACTCGGACACGTCTTTG	116
*IBSP*	F: ACGAACAAGGCATAAACGGCACCA R: CTTGCCCTGCCTTCCGGTCT	153
*E11* (*PDPN*)	F: TTACTAGCCATCGGCTTCATTG R: GGCGAGTACCTTCCCGACAT	71
*RANKL* (*TNFSF11*)	F: CTCAGCCTTTTGCTCATCTCACT R: CAAGAGGACAGACTCACTTTATGG	77
*DDC*	F: CAAGTCACTCCCGGCTGC R: CTCCTTCCCTCTCCTTCGGA	84
*ENO2*	F: GTGCACAGGCCAGATCAAGA R: ACAGCACACTGGGATTACGG	140
*RPL32*	F: GCACCAGTCAGACCGATATG R: ACTGGGCAGCATGTGCTTTG	151
*7SL*	F: ATCGGGTGTCCGCACTAAGTT R: CAGCACGGGAGTTTTGACCT	126

### Protein analysis

Analysis of the secretome of LNCaP, C4-2B, 2D hOB, hOBMT, LNCaP/hOBMT, and C4-2B/hOBMT was performed using a cytokine protein array (Profiler Human XL Cytokine Array Kit, ThermoFisher) according to the manufacturer’s instructions. Analysis was performed with two technical replicates and with cells from two donors, for 2D hOB, hOBMT, and co-cultures. Similar experimental design was used as for the RT-qPCR co-culture experiments On day 10, conditioned media and cell protein lysates were collected and analyzed. Protein arrays and westerns blots (WB) on conditioned media and WB on protein lysates were performed as detailed in the Methods Supplement.

### Statistical analysis

All statistical tests were performed in IBM SPSS Statistics 23 (IBM Corp). Significance level was determined *P < 0.05, **P < 0.01, ***P < 0.001, ****P < 0.000 1. Details are found in the Methods Supplement.

## Supplementary information

Supplemental Materials and Methods

Supplemental Figure S1

Supplemental Figure S2

Supplemental Figure S3

Supplemental Figure S4

Supplemental Figure S5

Supplemental Figure S6

Supplemental Figure S7

Supplemental Figure S8

Supplemental Figure S9

Supplemental Figure S10

Copyright Clearance

Supplementary Video
