# Supplementary material for: Engineering osteoblastic metastases to delineate the adaptive response of androgen-deprived prostate cancer in the bone metastatic microenvironment
Source: Bone Res. 2019 Apr 25;7:13. doi: 10.1038/s41413-019-0049-8 (PMC6486620; doi:10.1038/s41413-019-0049-8)
Supplement: Supplementary file 1 — Supplemental Materials and Methods [file 41413_2019_49_MOESM1_ESM.docx]

Engineering osteoblastic metastases to delineate the adaptive response of androgen-deprived prostate cancer in the bone metastatic microenvironment

Nathalie Bock^1,2,3^, Ali Shokoohmand^1,2,3,†^, Thomas Kryza^1,2,†^, Joan Röhl^1,2^, Jonelle Meijer^1,2,3^, Phong A. Tran^3,4^, Colleen C. Nelson^1,2^, Judith A. Clements^1,2,*^, Dietmar W. Hutmacher^1,2,3,4,5^*

1. School of Biomedical Sciences, Faculty of Health and Australian Prostate Cancer Research Centre (APCRC-Q), Institute of Health and Biomedical Innovation (IHBI), Queensland University of Technology (QUT), Brisbane, QLD, Australia
2. Translational Research Institute (TRI), QUT, Brisbane, QLD, Australia
3. Centre in Regenerative Medicine, IHBI, QUT, Kelvin Grove, QLD, Australia
4. Bone and Joint Disorders Program, School of Chemistry, Physics and Mechanical Engineering, Science and Engineering Faculty (SEF), QUT, Brisbane, QLD, Australia
5. Australian Research Council (ARC) Training Centre in Additive Biomanufacturing, QUT, Kelvin Grove, QLD, Australia

Correspondence to: [j.clements@qut.edu.au](mailto:j.clements@qut.edu.au), [dietmar.hutmacher@qut.edu.au](mailto:dietmar.hutmacher@qut.edu.au).

# **SUPPLEMENTAL MATERIALS AND METHODS**

## **3D scaffold manufacturing by melt electrowriting**

Microfiber scaffolds made of medical-grade polycaprolactone (mPCL, PURASORB PC12, 95-140 kDa, 2 mm pellets, Corbion Purac, The Netherlands) were manufactured by melt electrowriting (MEW) with an in-house-built apparatus (IHBI, QUT, Brisbane, Australia) and protocol described elsewhere.^1,2^ Briefly, mPCL was loaded into a 3 CC clear Luer-Lock plastic syringe (Nordson EFD, Australia) and pre-heated to 60 ºC overnight in a bench oven (Labec, Australia). The syringe was fitted with a 23 G tapered needle (Nordson EFD) and set in the MEW block heaters, at 74 ºC and 85 ºC for the syringe and needle block heaters, respectively. After 2 hours, the extrusion pressure was regulated to 2.2 bar and the working distance between the needle tip and aluminum collector set to 9 mm. A G-code was loaded in the Mach 3 software (Artsoft, USA). The voltage was increased to 10.1 kV and the print was started after 15 min from voltage stabilization, lasting 2.5 days until completion. The resulting printed scaffold had dimensions favorable to cell growth and infiltration (600 µm thickness, 12 µm fiber diameter, 150 µm pore size) and was laser-cut (ILS12.75, Universal Laser Systems Inc., USA) in 1×1 cm small scaffolds prior to chemical treatment.

## **G-code**

The following G-code (incremental type) was used to generate the layer-by-layer manufacturing of a microfiber polymeric scaffold with an overall size of 90 × 70 mm, spacing of 150 µm in between fibers, comprising 80 layers in the *z* direction in a ‘0 degree - 90 degree’ pattern, yielding approximately 600 µm in the *z* direction. In the following code, the sign ‘*’ indicates a new line in the code, i.e. all ‘*’ signs need to be replaced by pressing the ‘Enter’ key in the final code.

G-Code:

G17 G21 G40 G49 G54 G80 G94 F450 %G91 is relative coordinates! *F1000 *G91 *G1 x10 *M98 p1 l20 % trail print for jet stabilization *G1 y5 *M98 p1236 l40 % number of layers in overall scaffold construct--40X and 40Y *G1 x-10 **M30 **o1236 *M98 p1237 l300 % loop size/FD *G1 x90 *G1 y-90 *M98 p1238 l300 % loop size/FD *M99 **o1237 %x loops *G1 x90 *G1 y0.15 *G1 x-90 *G1 y0.15 *M99 **o1238 %y loops *G1 x-0.15 *G1 y90 *G1 x-0.15 *G1 y-90 *M99 **o1 % trail loop x100 *y0.2 *x-100 *y0.2 *M99 *M2.

## **3D scaffold surface modification**

The laser-cut 1×1 cm mPCL scaffolds were treated with sodium hydroxide (NaOH) and calcium phosphate (CaP) for hydrophilicity and osteoinductive properties, as described elsewhere.^3^ All chemicals mentioned below were obtained from Sigma-Aldrich, Australia.

### Surface activation

Scaffolds were immersed in 70% v/v ethanol under desiccator house-vacuum for 15 min. Scaffolds were rinsed twice with ddH_2_O and transferred in a pre-warmed (37 ºC) 2 M NaOH solution in a 50 mL reaction tube. After 5 min under desiccator house-vacuum, the tube was placed in a water bath at 37 ºC for 45 min. After NaOH solution removal, scaffolds were rinsed with ddH_2_O for eight washes, and pH was controlled to be neutral (7).

### Biomimetic mineralization

A 10× simulated body fluid solution (SBF 10×) was prepared by preparing *Solution A*, which involved dissolving the chemicals listed in [Supplemental Table 1](#_Supplemental_Table_1) in 300 mL ddH_2_O in a 500 mL beaker, under magnetic stirring. In a second beaker, 710 mg of disodium hydrogen phosphate (Na_2_HPO_4_) was dissolved in 15 mL ddH_2_O. Under magnetic stirring, the Na_2_HPO_4_ solution was added to *Solution A* dropwise, ensuring that pH remained at all times between 1.5 and 3.5 by HCl titration, until the final addition where pH reached 3.9. The final solution (SBF 10×) was topped-up to 500 mL with ddH_2_O (final pH of 4). A 50 mL aliquot was placed in a 50 mL beaker and small amounts of sodium hydrogen carbonate (NaHCO_3_) powder were gradually added until pH reached 6. The solution was 0.2 µm-filtered and placed in a 50 mL reaction tube with the mPCL scaffolds. After 5 min under desiccator house-vacuum, the tube was placed in a water bath at 37 ºC for 30 min. A new 50 mL SBF aliquot was prepared and the step was repeated once more. After rinsing once with ddH_2_O, scaffolds were immersed in a pre-warmed (37 ºC) 0.5 M NaOH solution. After 5 min under desiccator house-vacuum, the tube was placed in a water bath at 37 ºC for 30 min. Scaffolds were washed five times with ddH_2_O until pH was neutral. Scaffolds were dried overnight in the desiccator and scanning electron microscopy was performed for quality control. The final treated scaffolds are referred to as ‘CaP-mPCL’ scaffolds.

**Supplemental Table 1**. Reagent amounts and order of use for simulated body fluid (SBF) 10×.

| Reagent | Amount | Order |
| --- | --- | --- |
| Sodium chloride, NaCl | 29.215 g | 1 |
| Potassium chloride, KCl | 0.187 g | 2 |
| Calcium chloride dehydrate, CaCl_2_·2H_2_O | 1.838 g | 3 |
| Magnesium chloride hexahydrate, MgCl_2_·6H_2_O | 0.508 g | 4 |

### Sterilization

Prior to cell seeding, CaP-mPCL scaffolds were immersed in 70% v/v ethanol in Petri dishes in a biological safety cabinet class II (laminar flow), under aseptic conditions. After 20 min, ethanol was removed and the scaffolds were let to dry overnight inside the laminar flow cabinet. Scaffolds were exposed to the UV light from the cabinet for 20 min on each side and kept in the Petri dishes prior to cell seeding.

## **Primary cell isolation from bone tissue**

Human primary osteoprogenitor cells were isolated from bone tissue obtained under informed consent from male donors undergoing hip and knee replacement surgery (QUT ethics approval number 1400001024), as described previously.^4^ Non-sclerotic, trabecular bone was collected either from the tibial plateau/femoral condyles from the knee or from the acetabular ground from the hip. Bone fragments (4-5 mg) were minced and washed in sterile phosphate buffer saline (PBS) without divalent ions (Gibco, Australia). Bone fragments were transferred to 175 cm^2^ cell culture-treated flasks with 18 mL of growth media (GM), containing α-MEM with ribonucleosides, deoxyribonucleosides, phenol Red and L-glutamine (cat.12571), with 10% fetal bovine serum (FBS) and 1% penicillin/streptomycin (P/S, 10,000 U/mL stock solution), all from Gibco. Osteoprogenitor cell outgrowth occurred after 7-10 days. Cells were collected using 0.25% w/v Trypsin – 1 mM EDTA (Gibco) at 80% confluence, and further expanded using GM. Cells from passages 3-4 were used for culture within the CaP-mPCL scaffolds and were tested free of mycoplasma. Three different healthy male donors were used, ranging from 65 to 79 year of age.

## **Bioengineering of human osteoblast-derived microtissues**

The sterilized CaP-mPCL scaffolds were placed in cell-culture treated 24-well plates. Primary osteoprogenitor cells were seeded at a density of 0.4×10^6^ cells/scaffold. Briefly, cells were first concentrated at 8×10^6^ cells/mL in GM. Five drops of 10 µL of cell suspension were seeded in each corner and in the center of each scaffold (total volume of 50 µL/scaffold). Cell constructs were incubated for 1 hour in a humidified incubator (37 ºC, 95% air, 5% CO_2_) before 50 µL of GM was added in the center of the scaffold. After 3 hours, 2 mL of GM was added to each well with media changes every 2-3 days. After 14 days, the cellular constructs were transferred to new 12-well plates each with 2 mL of osteogenic media (OM), containing GM + 10 mM β-glycerophosphate, 0.17 mM ascorbic acid, 100 nM dexamethasone (all from Sigma-Aldrich, Australia). Media was changed every 3-4 days and the resulting cellular microtissues were cultured for either 4, 7, 10 or 13 weeks, to study mineralization. According to the mineralization experiment, for further experiments, the microtissues were mostly cultured for 10 weeks in OM, yielding an optimal microtissue with extensive bone ECM deposition. The final bioengineered constructs are hereafter referred to as human osteoblast-derived microtissues (hOBMT).

## **Scanning electron microscopy**

The morphology of the hOBMT and co-culture microtissues were assessed by scanning electron microscopy (SEM). All chemical were obtained from Sigma-Aldrich. At desired time points, the microtissues were fixed with 3% glutaraldehyde, incubated in 0.1 M sodium cacodylate buffer for 30 min, and immersed in 1% osmium tetroxide in cacodylate buffer for 1 hour at room T. After two washes with ultra-pure water (10 min each), samples were dehydrated through a graded series of ethanol (ethanol 50% v/v for 10 min, twice, ethanol 70% v/v for 10 min, twice, ethanol 90% v/v for 10 min, twice, ethanol 100% v/v for 15 min, twice). After final incubation in hexamethyldisilazane (HMDS) for 30 min, twice, the dehydrated microtissues were mounted on aluminum stubs and gold-coated for 225 seconds at 30 mA (Leica EM SCD005, Leica Microsystems, Australia). The microtissues were imaged with a SEM (FEI Quanta 200 Environmental SEM, FEI, The Netherlands) operating at an accelerating voltage of 5 kV in high vacuum mode, at a 7.4 mm working distance.

## **3D mineralization**

To quantify *in vitro* mineralization, hydroxyapatite (HA) deposition within the hOBMT was measured after 4, 7, 10 and 13 weeks in culture in OM, using the OsteoImage mineralization assay (Lonza, Australia) as per the manufacturer’s protocol. Briefly, at each time point, the hOBMT were washed twice with PBS for 10 min and fixed with methanol for 1 hour. After fixation, 4 mm discs were collected from the hOBMT using biopsy punches, and washed two times with the supplied wash buffer. The hOBMT disc samples were placed in a 96-well plate and stained with the supplied staining reagent (0.1 mL/well) at room T for 30 min, protected from light, allowing mineralized HA nodules to be stained with the fluorescent dye. After 3 washes in wash buffer (10 min each wash), the scaffolds were imaged using a spectral spinning disc confocal (SDC) microscope (X-1 Yokogawa spinning disc with Borealis modification, fitted with 4× and 20× objectives operating with the green (ex 488 nm) filter set). For 3D quantification, *z*-stacks were obtained using the 20× objective, with 2.5 µm as the step size and 50 µm thickness (21 images). Maximum intensity projections were produced from the z-stacks and positive signal was determined using ImageJ analysis software (version 1.51j8, National Institute of Health (NIH), USA)^5^ with 8 replicates on average per condition, for 2 donors.

## **Immunofluorescence**

All chemicals were purchased from Sigma-Aldrich. Upon culture, the media was aspirated and the hOBMT were washed with PBS twice. The microtissues were fixed in 4 % paraformaldehyde (PFA, Sigma-Aldrich) for 40 min at room T, and washed in PBS two times (10 min each), before permeabilization in 0.2 % Triton X-100 in PBS for 5 min at RT. After two washes in PBS (5 min each), samples were transferred in 0.5 % bovine serum albumin (BSA) in PBS for at least 10 min. Primary antibody solutions in 0.5 % BSA/PBS (details outlined in Supplemental Table 2), were used on the hOBMT (50 µL/microtissue) overnight at 4 ºC. After three PBS washes (10 min each), the samples were incubated with secondary antibody solutions in 0.5 % BSA/PBS, containing FITC-conjugated phalloidin (200 U/mL) and DAPI (5 µg/mL) for 1 hour. After three washes in PBS (10 min each), fresh PBS with added. The plate was covered by foil and kept at 4 ºC until imaging.

**Supplemental Table 2**. Antibodies and antigen retrieval details for immunohistochemistry (IHC) and immunofluorescence (IF)

| Antibody | Company | Product | IHC - Antigen Retrieval | IHC Dilution and Incubation | IF Dilution and Incubation |
| --- | --- | --- | --- | --- | --- |
| Mouse anti-human type I collagen | Abcam | ab23446 | Proteinase K, 15 min at RT | 1:100 o/n 4 °C | 1:100 o/n 4 °C |
| Mouse anti-human osteocalcin | Abcam | ab13418 | Proteinase K, 15 min at RT | 1:300 o/n 4 °C | 1:100 o/n 4 °C |
| Rabbit anti-human osteopontin | Abcam | ab8448 | Proteinase K, 15 min at RT | 1:500 1h RT | N/A |
| Rabbit anti-human fibronectin | Abcam | ab23446 | Tri-sodium Citrate Buffer, pH 6.0 (95 °C/4 min) | 1:1000 1h RT | N/A |
| Rabbit anti-human sclerostin | Abcam | ab63097 | Proteinase K, 10 min at RT | 1:300 1h RT | 1:100 o/n 4 °C |
| Mouse anti-human podoplanin (E11) | Abcam | ab10288 | Tri-sodium Citrate Buffer, pH 6.0 (95 °C/4 min) | 1:200 1h RT | N/A |
| Mouse anti-human DMP-1 | Gift from Prof. Yin Xiao (QUT) | N/A | Tri-sodium Citrate Buffer, pH 6.0 (95 °C/10 min) | 1:100 1h RT | N/A |
| Alexa Fluor 488 phalloidin | Invitrogen | A12379 | N/A | N/A | 1:250 1h RT |
| 4',6-diamidino-2-phenylindole (DAPI) | Sigma-Aldrich | D9542 | N/A | N/A | 1:1000 1h RT |
| Goat anti-mouse IgG (H+L) cross-adsorbed 2ndary antibody, Alexa Fluor 488 | Invitrogen | A11001 | N/A | N/A | 1:200 1h at RT |
| Goat anti-rabbit IgG (H+L) cross-adsorbed 2ndary antibody, Alexa Fluor 488 | Invitrogen | A11008 | N/A | N/A | 1:200 1h at RT |

## **Immunohistochemistry**

Upon culture, media was aspirated and the hOBMT were washed with PBS twice. The hOBMT were fixed in PFA for 40 min and washed twice in PBS for 20 min. Then microtissues were dehydrated using an automated histology tissue processor (Excelsior ES, Thermo Scientific, Australia) before embedding in paraffin wax (Sigma-Aldrich) in histology aluminum molds. Serial histology sections (5 µm) were used for hematoxylin and eosin (H&E) staining and immunohistochemistry using various antibodies, as outlined in Supplemental Table 2. Sections were deparaffinized and rehydrated before antigen retrieval. The sections were either incubated with Proteinase K (Dako, Denmark) for 10 min at RT or heated in a decloaking chamber (Biocare Medical, Australia) at 95 ºC for 4 min (Biocare Medical, Australia) with tri-sodium citrate buffer (pH = 6). After washing with 50 mM Tris-HCl buffer twice, endogenous peroxidase activity was quenched with 3% hydrogen peroxide (Sigma-Aldrich) for 15 min and non-specific binding sites were blocked with a blocking buffer (2% BSA in PBS) for 1 hour. Sections were incubated with primary antibodies diluted in the blocking buffer. After three washes in Tris-HCl, the sections were incubated with the mouse or rabbit secondary antibodies (DAKO Envision + Dual System HRP (Dako, Australia)) for 30 min at room T. After 2 washes in Tris-HCl, a few drops of DAB solution (DAKO liquid DAB + Substrate Chromogen System (Dako, Australia)) were pipetted onto the sections. As soon as the color changed, sections were immersed in tap water, followed by Tris-HCl. The sections were counter-stained with Mayer’s Haematoxylin (Sigma-Aldrich) before dehydration and mounting using Pertex mounting media (Sigma-Aldrich). Images were captured using a high throughput slide scanner (Leica SCN400, Leica Microsystems, Australia).

**Microscopy**

Most static 3D imaging was done using a Nikon spectral spinning disc confocal microscope (SDC, X-1 Yokogawa spinning disc with Borealis modification) fitted with either a Plan Fluor 4×, Plan Apo 10×, Plan Fluor ELWD 20× DIC or Plan Fluor 40× DIC objectives. Specific *z*-stack details are reported individually for each characterization. Routine fluorescence imaging was done using an IX73 Olympus inverted fluorescence microscope equipped with either a DP72 camera or XM10 camera. Green (ex 488 nm), red (ex 561 nm) and blue (ex 405 nm) filter sets were used, where applicable.

## **Physicochemical characterization**

The physicochemical characterization of the hOBMT was investigated using energy dispersive x-ray spectroscopy (EDS) and SEM (Zeiss FESEM, Germany) at an accelerating voltage of 20 kV. All the microtissues were fixed with 4% PFA for 40 min, and dehydrated as mentioned previously in the SEM section, before carbon coating. Images were acquired with a Sigma VP Field Emission SEM operating at 10 kV (Zeiss, NSW, Australia) in secondary electron (SE) mode and back scattered electron mode (BSE) for visualization of both topography and density in a single image. Images were colored post-process by stacking to a single RGB image using ImageJ with the SE micrograph assigned to the green channel and the BSE micrograph to the red channel. The red/orange and green colors indicated denser and less dense material, respectively. EDS was acquired by point-analysis of at least eight random spots per biological replicate.

## **Cell viability, DNA content, metabolic activity and alkaline phosphatase expression**

### Cell viability

Cell viability in the hOBMT was measured using a Live/Dead staining assay with fluorescein diacetate (FDA) and propidium iodide (PI, Invitrogen, Australia). The microtissues were washed twice with PBS, followed by incubation in FDA (0.67 µg/mL) and PI (5 µg/mL) solution (1 mL) for 5 min at 37 ºC in the dark. After washing with PBS, the specimens were immediately imaged using SDC fitted with either a Plan Fluor 4× or Plan Apo 10×, using the green (ex 488 nm) and red (ex 561 nm) filter sets, for live and dead cells, respectively. Maximal intensity projections were made from *z*-stacks using the 10× objective, with 2.5 µm as the step size and 100 µm thickness.

### DNA content

For cellular DNA content analysis, the hOBMT were frozen at -80 °C for at least 48 hours after two washes in PBS (20 min each). Next, the microtissues were placed in 1.5 mL reaction tubes containing 500 µL of Proteinase K (Invitrogen, Australia) dissolved in phosphate buffered EDTA (PBE) at 0.5 mg/mL, and heated at 60 °C for 12 h using a block heater. The solution was diluted at a ratio of 1/50 in PBE, and dispensed in triplicates (100 µL) into black 96-well plates (Corning, Australia), and PicoGreen dsDNA quantitation (Invitrogen, Australia) working solution (100 µL) was added. After 5 min of incubation in the dark, the fluorescence (excitation 485 nm, emission 520 nm) was measured using a FLUOstar Omega plate reader (BMG LABTECH, Australia). A standard curve of known λ DNA concentrations ranging from 10 ng/mL to 1 µg/mL was used to calculate the final DNA content of the samples.

### Metabolic activity

Metabolic activity was measured using the PrestoBlue cell viability assay (Invitrogen, Australia). Microtissues were incubated with their respective media, containing 10% v/v PrestoBlue cell viability assay for 3 hours in a humidified incubator (37 ºC, 95% air, 5% CO_2_). Empty scaffolds with the corresponding media were used as negative controls. Upon incubation, the solutions were transferred into black 96-well plates (Corning, Australia). Fluorescence (excitation 544 nm, emission 590 nm) was determined using a FLUOstar Omega plate reader (BMG LABTECH, Australia) and corrected with negative control background.

### Alkaline phosphatase activity

The expression of Alkaline Phosphatase (ALP) in medium was measured using the SigmaFAST kit (Sigma-Aldrich), as per the manufacturer’s protocol. Briefly, the samples were immersed three times in phenol-free α-MEM (with ribonucleosides, deoxyribonucleosides, phenol Red and L-glutamine, cat.12571, Gibco, Australia) for 20 min each time. Culture media was pipetted in triplicate in a 96-well plate (100 µL) and P-Nitrophenyl phosphate (pNPP) in Tris-base buffer (100 µL) was added to each well, and

incubated for another 24 hours. Negative controls consisted of fresh media + pNPP. At the end of the second incubation period, the plate was brought back to ambient temperature (20 °C) for 15 min and the absorbance was read at 405 nm using a plate reader (Benchmark PlusTM microplate spectrophotometer, BIO RAD) and corrected with negative control background.

## **Cancer cell attachment assay**

Cancer cell attachment to hOBMT in either PCa-Norm (RPMI, 5% FBS) or PCa-AD (RPMI, 5% CSS) media was measured as follows. Mature hOBMT were sectioned as 5 mm tissue samples using biopsy punches and placed onto 24-well plates coated with 1% Agar (Sigma-Aldrich). Six replicates coming from different hOBMT were used per condition. LNCaP, C4-2B and PC3 were pre-conditioned in PCa-Full or PCa-AD media for 5 days prior to the attachment assay. A concentration of 2×10^5^ cell/mL was prepared for the three cell types and two conditions, and 500 µL of the cell suspensions was added to each well. Wells that contained no hOBMT were used as controls. Co-cultures were transferred onto a rocking mixer platform set at 80 oscillations per min inside the incubator (RPM4, Ratek Laboratory Equipment) for 24 hours. The supernatants and washing media were collected (3.5 mL total) and centrifuged at 300 g for 3 min at RT. Cancer cell attachment on hOBMT ($\bar{{Att}_{hOBM}})$ was measured according to the formula below, with $\bar{C_{ctrl}}$ as the average cell concentration value from control wells, $\bar{C_{hOBM}}$ as the average cell concentration value collected per well, and n the number of wells. Results are expressed as box-and-whisker plots for 3 independent experiments.

$$\bar{{Att}_{hOBM}} (\%)=\frac{\sum_{n} \frac{\bar{C_{ctrl}}-\bar{C_{hOBM}}}{\bar{C_{ctrl}}}}{n}$$

**Morphometric Analysis**

For morphometric analysis, automated surface statistics were computed from *z*-stacks in Imaris (algorithm parameters: surface area detail 1 μm, threshold: automatic, diameter 11 μm, quality filter: automatic). For ImageJ processing, maximum projections were made from the *z*-stacks. Following a Gaussian blur filter (value: 2) and Huang thresholding, the angle value for each particle was obtained. For each field of view, the standard deviation (SD) from all angles was measured. The average of all SD values per condition was presented as the degree of cancer cell orientation onto the hOBMT. At least 2 hOBMT/condition were analyzed with more than three fields of view, generating an average of 335 cells analyzed per condition. This was done for two independent experiments and for the hOBMT from two donors.

**Migration and proliferation analysis**

For migration analysis (mean square displacement, cellular speed, track length and straightness), automated spots statistics were computed from live cell imaging series using Imaris (algorithm parameters: estimated cell diameter 18 μm, intensity filter 30-230, max distance jumps 20 μm, max gap size 5) using Imaris. For proliferation, live cell image series were analyzed using ImageJ. Following a Gaussian blur filter (value: 2) and Huang thresholding for the mKO2 (red) signal, the area occupied by PCa cells at each time point was measured using the area measurement function of ImageJ. An average of 8 fields of view were recorded per co-culture microtissue and 2 microtissues/condition from 2 independent experiments were used, generating an average of 270 tracks analyzed per condition.

## **Prostate cancer cell (PCa)/hOBMT co-culture for gene and protein analysis**

PCa/hOBMT were co-cultured after 10 days with either LNCaP or C4-2B in either PCa-AD or PCa-DHT media and compared with their mono-cultures counterparts (LNCaP or C4-2B only and hOBMT only), cultured in the same conditions. Specifically, cancer cells were seeded as previously mentioned (5×10^4^ cell/scaffold), on day 0, with PCa-Norm medium. LNCaP and C4-2B controls were seeded on 6-well-plates at half the scaffold seeding density (2.5×10^4^ cell/well). Unattached PCa cells were removed after 24 hours of co-culture and fresh PCa-Norm media was added. On day 3, media was changed to either PCa-AD media (10% CSS instead of FBS), or PCa-DHT media (10% CSS instead of 10% FBS, + 10 nM DHT). Media was changed every second day. On day 10, RNA was extracted, reverse transcribed, and processed for RT-qPCR as detailed below. Expression of target mRNA was determined using the delta-delta Cq method, using 7SL and RPL32 gene expression, geometrically averaged, as reference genes. For protein analysis, similar experimental design was used except that on the last media change at day 8, serum-free PCa-AD and PCa-DHT were used. GADPH was used as the reference protein. Results are expressed as means ± standard error (SE) from three independent experiments/donor.

## **RNA isolation, reverse transcription and quantitative PCR**

RNA was collected using 1 mL of TRIzol reagent (Thermo Fisher, Australia) on ice. Mono-cultures were scraped using cell scrapers, and microtissues were manually sectioned with sterilized surgery scissors to 0.5 mm pieces, while soaking in Trizol. Trizol extracts were transferred to 2 mL DNase/RNase-free tubes and placed 20 min on ice prior to storage in -80 ºC overnight. Next, tubes were thawed on ice and centrifuged at 15,000 rpm for 10 min at 4 ºC. Supernatants were transferred to new DNase/RNase-free 2 mL tubes and kept on ice. RNA was extracted using Direct-zol RNA MiniPrep (cat. R2050, Zymo Research, Australia), according to the manufacturer’s instructions, including DNase I treatment (0.375 U/µL, 80 µL) for 15 min at room T. Purified RNA concentrations were determined using a Nanodrop spectrophotometer (Thermo Fisher, Australia) and stored at -80 ºC until use. All samples used for reverse transcription (RT) had 260/280 nm and 260/230 nm ratios within 1.95-2.0 and 2.0-2.2, respectively. RT was done using SensiFAST cDNA Synthesis Kit (Bioline, Australia), as per the manufacturer’s protocol, using 410 ng of RNA for experiments shown in Fig. 1g-h, Fig. S1d-e and Fig. 2e-f, and 437 ng of RNA for experiments shown in Fig. 3c-d, Fig. S3d, Fig. 6 and Fig. S8. Briefly, RNA samples (ranging from 2 to 15 µL volume), were mixed with 4 µL of 5x TransAmp Buffer, 1 µL of Reverse Transcriptase solution, and topped up to 20 µL with ultra-pure water (Thermo Fisher). Using a thermal cycler (Vapo.protect^TM^, Eppendorf), RNA was converted to cDNA using the following sequence: 25 ºC for 10 min, 42 ºC for 15 min, 85 ºC for 5 min and held at 4 ºC. cDNA products were diluted 1:3 and 1:5 in ultra-pure water and stored at -80 ºC until use. Quantitative RT-PCR (RT-qPCR) was carried out in triplicates in 384-well plates, using per well; 5 µL of SYBR Green PCR master mix (Thermo Fisher), 2 µL of sample (either 13.6 ng of cDNA for experiments shown in Fig. 1g-h, Fig. S1d-e and Fig. 2e-f or 8.8 ng of cDNA for experiments shown in Fig. 3c-d, Fig. S3d, Fig. 6 and Fig. S8), 0.5 µL of 10 µM forward and reverse specific primers (BLAST validated, Sigma-Aldrich, Table 1), and 2.5 µL of ultra-pure water. ‘No template’ controls were added on all plates and used as negative controls. RT-qPCR was performed on a ViiATM 7 Real-Time PCR System (Thermo Fisher) with the following conditions: 95.0 °C for 10 min, 40 cycles of 95.0 °C for 15 s, and 60 °C for 1 min. Melt curves were obtained with an extra 95.0 °C for 15 s, 60 °C for 1 min and 95.0 °C for 15 s. Data analysis was done using QuantStudio Real-Time PCR software (Life Technologies, Australia). Amplification specificity was verified by melting curve analysis. The ‘no template’ controls were either ‘undetermined’ or expressed after a minimum of 4 more cycles compared to any other sample. Expression of target mRNA was determined using the delta-delta Cq method, using 7SL and RPL32 gene expression, geometrically averaged^6^, as reference genes. Results were compared to conditions mentioned individually in the legends of each figure.

## **Protein arrays**

After 10 days co-culture, conditioned media was collected and pulled together for each condition and each patient (2.5 mL collected per condition and per patient). Protein concentrations were measured by the bicinchoninic acid assay (BCA, Thermo Fisher) and ranged from 249 to 350 µg/mL. Conditioned media were diluted to equivalent amount among samples. A total of 1.5 mL was used for analysis by a cytokine protein array (Profiler Human XL Cytokine Array Kit, Thermo Fisher) used according to the manufacturer’s instructions. Membranes were exposed to X-ray for 5 min (ChemiDoc XRS+ with ImageLab 5.1 Software, Bio-Rad) and quantitatively analyzed using ImageJ. Results were expressed as the means of relative intensity (%) of duplicate spots relative to the mean intensity of three positive control spots of each array.

## **Western blots**

Whole-cell proteins were extracted on ice using radio-immunoprecipitation assay (RIPA) lysis buffer (150 mM sodium chloride, 1.0% Triton X-100, 0.5% sodium deoxycholate, 0.1% SDS, 1 mM sodium orthovanadate, 1 mM sodium fluoride, 50 mM Tris, pH 8.0, with a protease inhibitor cocktail (Sigma-Aldrich). An equal amount of protein measured with the Micro BCA Protein Assay Kit (Thermo Fisher‎) was separated by sodium dodecyl sulfate-polyacrylamide gel electrophoresis (SDS-PAGE) using SDS-PAGE buffer (14.4% glycine, 100 mM dithiothreitol, 10% glycerol, 2% SDS) and transferred onto methanol-activated polyvinylidene difluoride (PVDF) membranes by liquid transfer. After blocking, PVDF membranes were blocked for 1 hour in TBS-T (TBS + 0.1% Tween-20) containing 5% skim milk and then incubated with respective primary antibodies diluted in TBS-T containing 5% skim milk overnight at 4 °C, followed by incubation with species-appropriate HRP conjugated secondary antibodies for 90 min. After submerging membranes for 10 min in Pierce ECL Western Blotting Substrate (Thermo Fisher), membranes were exposed using the ChemiDoc Touch Imaging System (Bio Rad, Australia). Consistent protein loading and transfer was determined by reprobing membranes with either an anti-actin or anti-tubulin antibody. Densitometry analysis was carried out using ImageJ based on the images from 3 independent experiments.

## **Long term morphology of metastatic microtissues**

For long-term morphology of the PCa/hOBMT co-cultures, samples were collected after 7 and 21 days of co-culture in PCa-Full or PCa-AD media for SEM imaging, IF and IHC staining as per the methods above. After immunostaining with DAPI and phalloidin, a SDC microscope (X-1 Yokogawa spinning disc with Borealis modification) was used to collect *z*-stacks of the co-culture microtissues fitted with a 10× Plan Apo objective. Fluorescence was recorded in the red (ex 561 nm), green (ex 488 nm) and blue (ex 405 nm) channels. Maximal intensity projections were made from z-stacks using 1 µm as the step size and 70 µm thickness.

## **Statistical analysis**

All statistical tests were performed in IBM SPSS Statistics 23 (IBM Corp). For biological and biochemical experiments, unless otherwise stated, three independent experiments (from the same donor) were conducted with means ± standard errors presented. For comparison between 2 groups and n < 4/group, Mann-Whitney non-parametric test was done on medians. When more than two groups were compared, and Levene’s *p* > 0.05, 1-way ANOVA was used with Tukey Post hoc test, while Games-Howell was used for a Levene’s *p* < 0.05. For more than two groups and more than two variables, a general linear model (univariate analysis) was used with Tukey Post hoc test, performed when overall significance was met.

**References**

1 Brown, T. D. et al. Design and fabrication of tubular scaffolds via direct writing in a melt electrospinning mode. Biointerphases 7, 13, doi:10.1007/s13758-011-0013-7 (2012).

2 Martine, L. C. et al. Engineering a humanized bone organ model in mice to study bone metastases. Nature Protocols 12, 639-663, doi:10.1038/nprot.2017.002 (2017).

3 Vaquette, C., Ivanovski, S., Hamlet, S. M. & Hutmacher, D. W. Effect of culture conditions and calcium phosphate coating on ectopic bone formation. Biomaterials 34, 5538-5551, doi:10.1016/j.biomaterials.2013.03.088 (2013).

4 Reichert, J. C. et al. Mineralized human primary osteoblast matrices as a model system to analyse interactions of prostate cancer cells with the bone microenvironment. Biomaterials 31, 7928-7936, doi:10.1016/j.biomaterials.2010.06.055 (2010).

5 Schneider, C. A., Rasband, W. S. & Eliceiri, K. W. NIH Image to ImageJ: 25 years of image analysis. Nat. Methods 9, 671-675, doi:10.1038/nmeth.2089 (2012).

6 Vandesompele, J. et al. Accurate normalization of real-time quantitative RT-PCR data by geometric averaging of multiple internal control genes. Genome Biol 3, Research0034 (2002).
