# Supplementary figures and images for: Engineering osteoblastic metastases to delineate the adaptive response of androgen-deprived prostate cancer in the bone metastatic microenvironment
Source: Bone Res. 2019 Apr 25;7:13. doi: 10.1038/s41413-019-0049-8 (PMC6486620; doi:10.1038/s41413-019-0049-8)

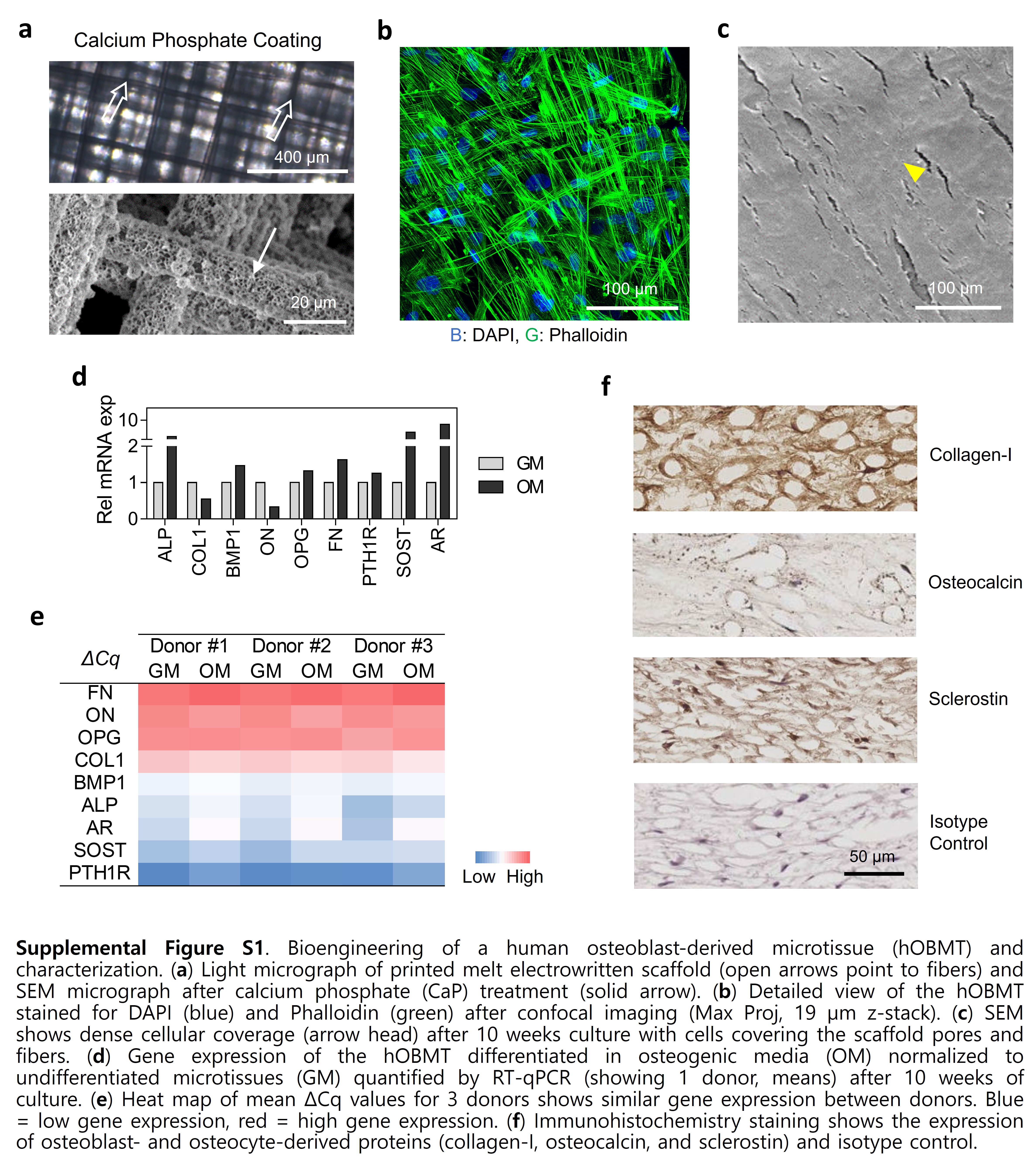

Supplement: Supplementary file 2 — Supplemental Figure S1 [file 41413_2019_49_MOESM2_ESM.jpg]

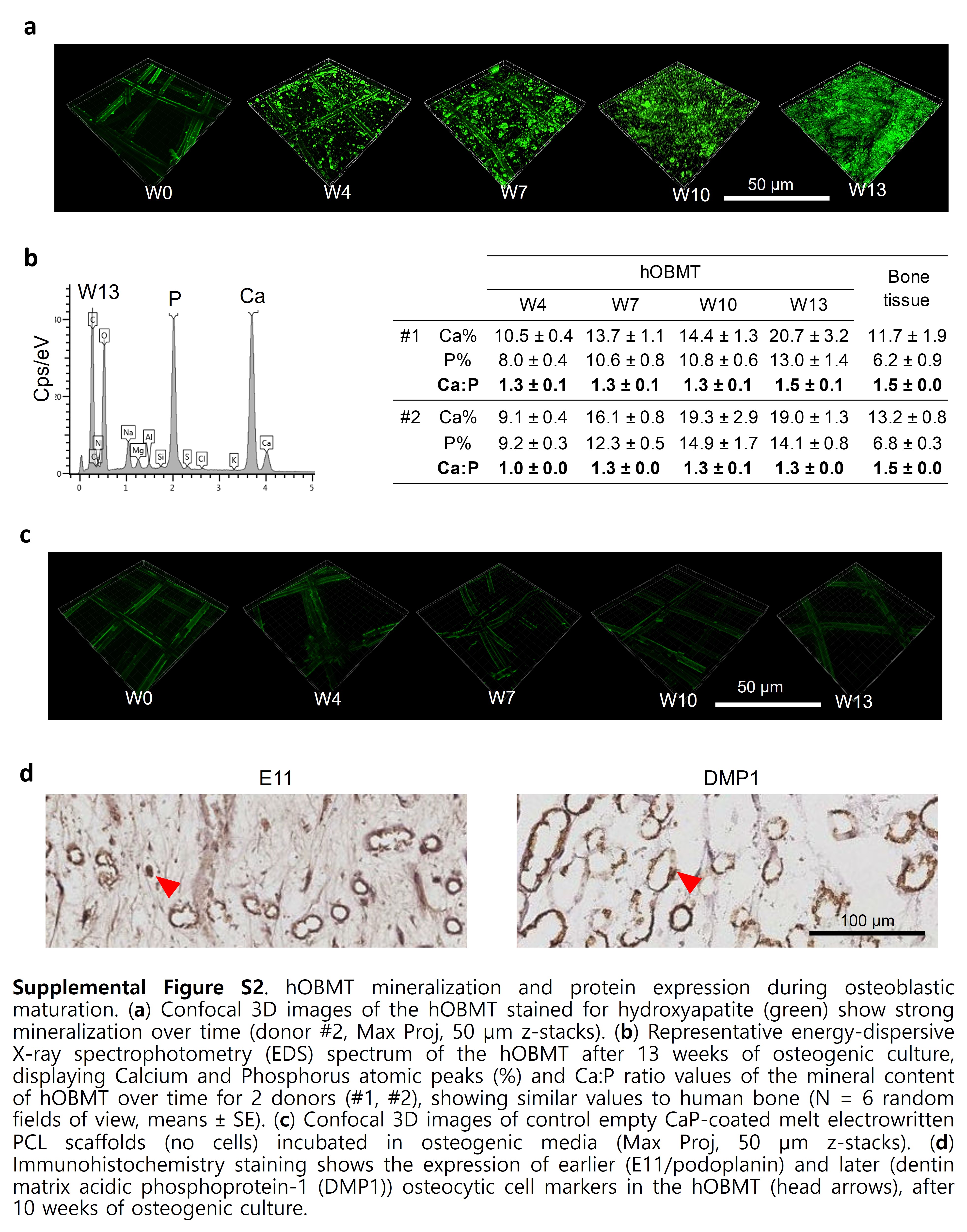

Supplement: Supplementary file 3 — Supplemental Figure S2 [file 41413_2019_49_MOESM3_ESM.jpg]

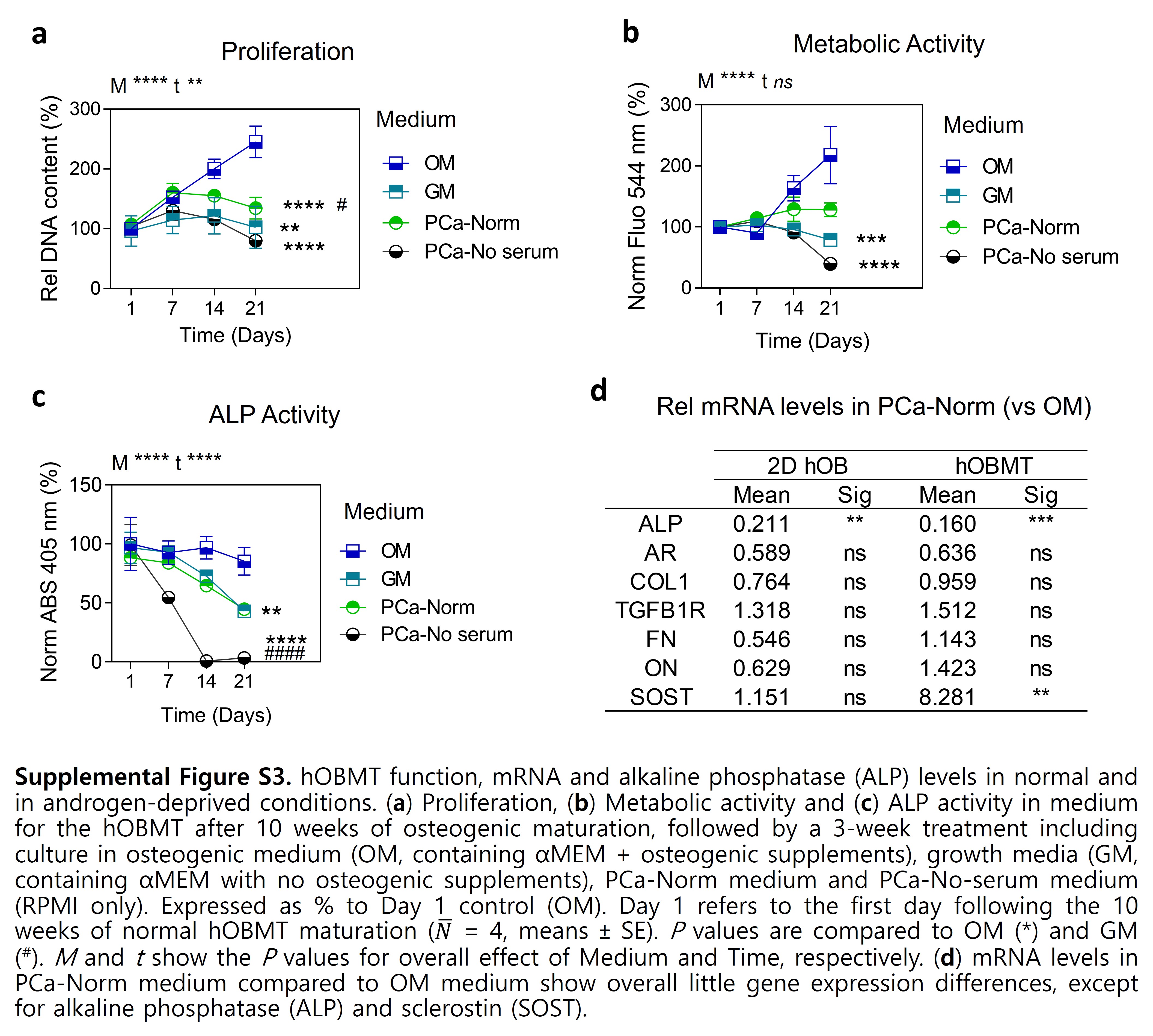

Supplement: Supplementary file 4 — Supplemental Figure S3 [file 41413_2019_49_MOESM4_ESM.jpg]

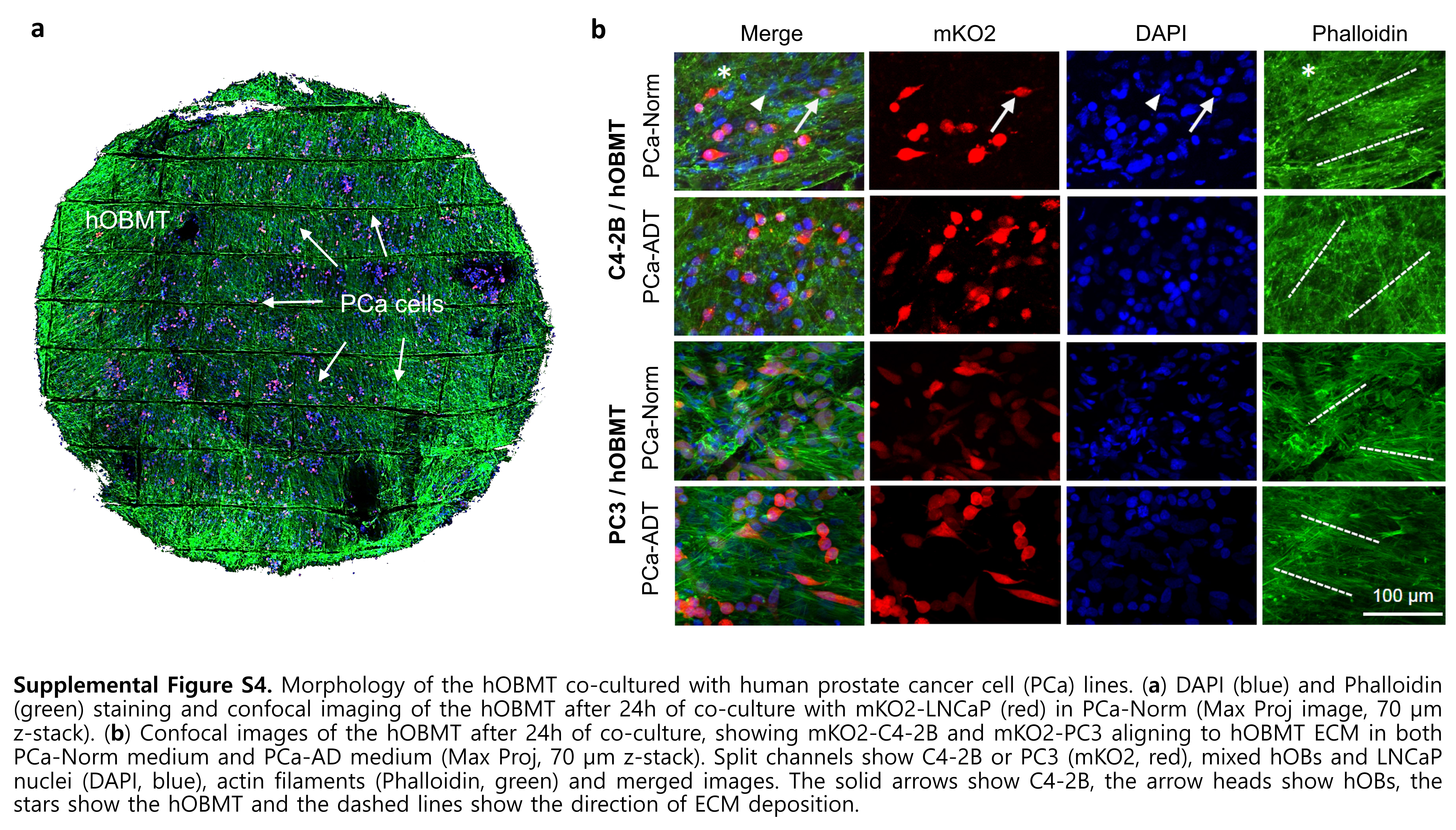

Supplement: Supplementary file 5 — Supplemental Figure S4 [file 41413_2019_49_MOESM5_ESM.jpg]

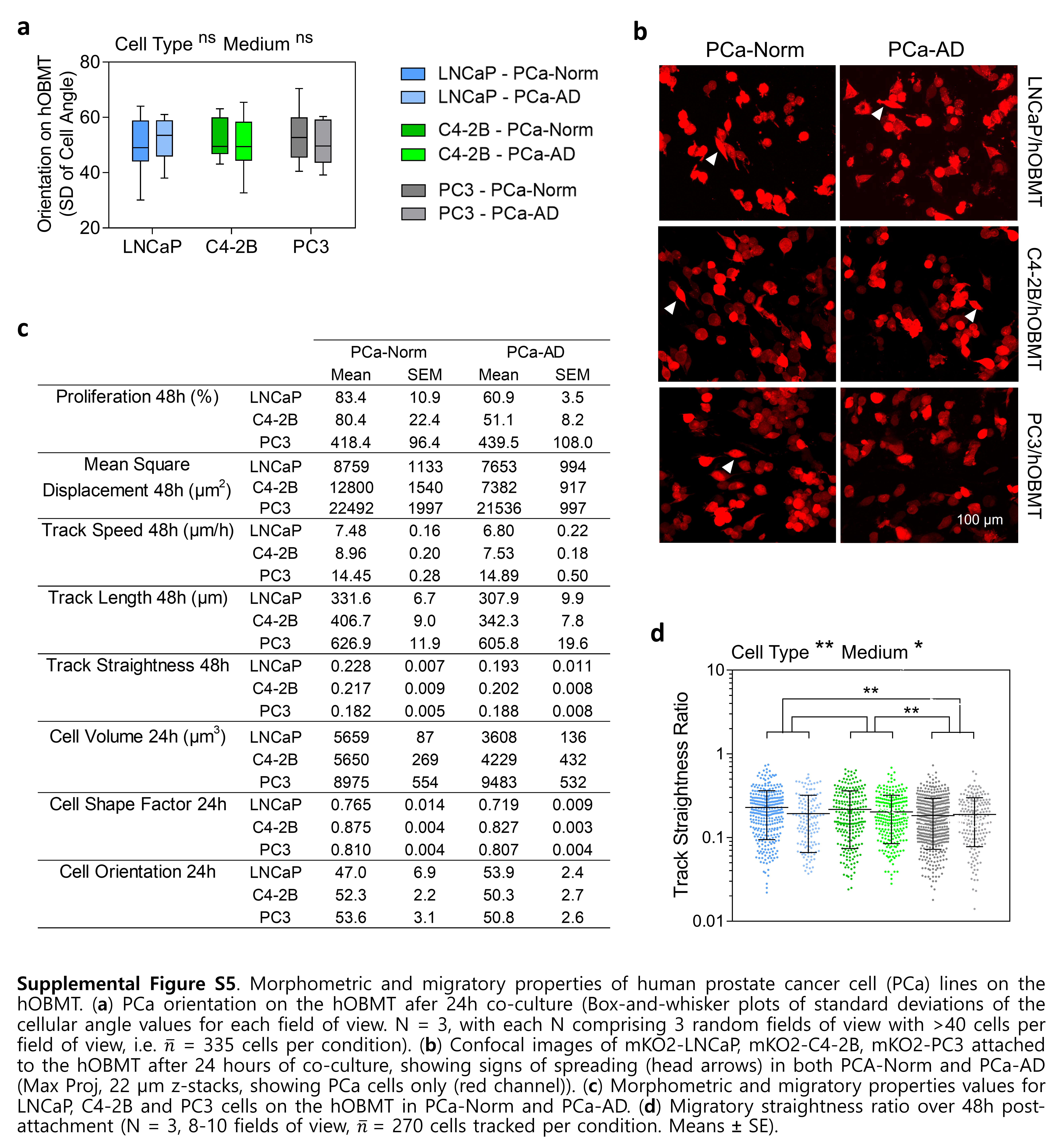

Supplement: Supplementary file 6 — Supplemental Figure S5 [file 41413_2019_49_MOESM6_ESM.jpg]

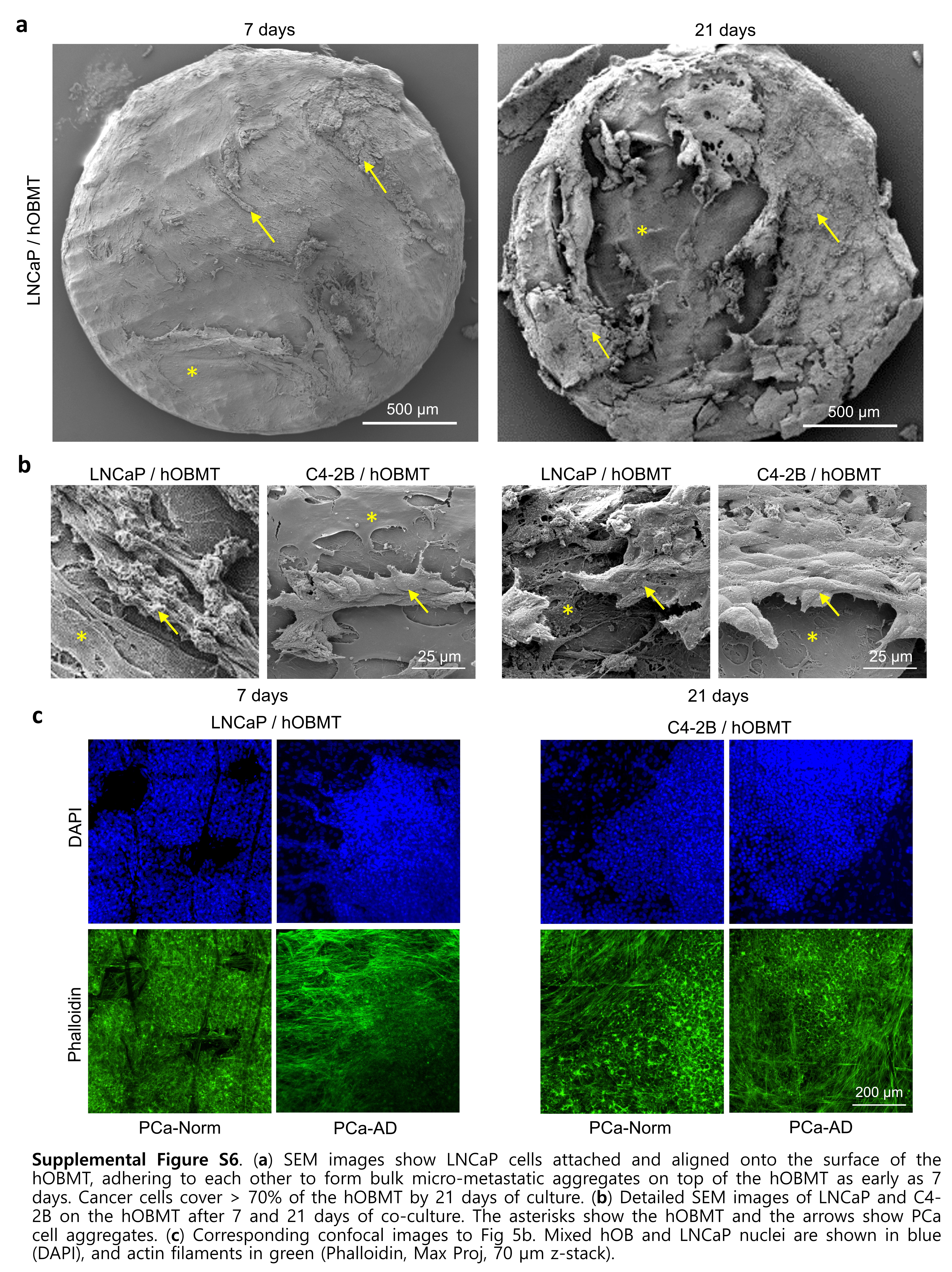

Supplement: Supplementary file 7 — Supplemental Figure S6 [file 41413_2019_49_MOESM7_ESM.jpg]

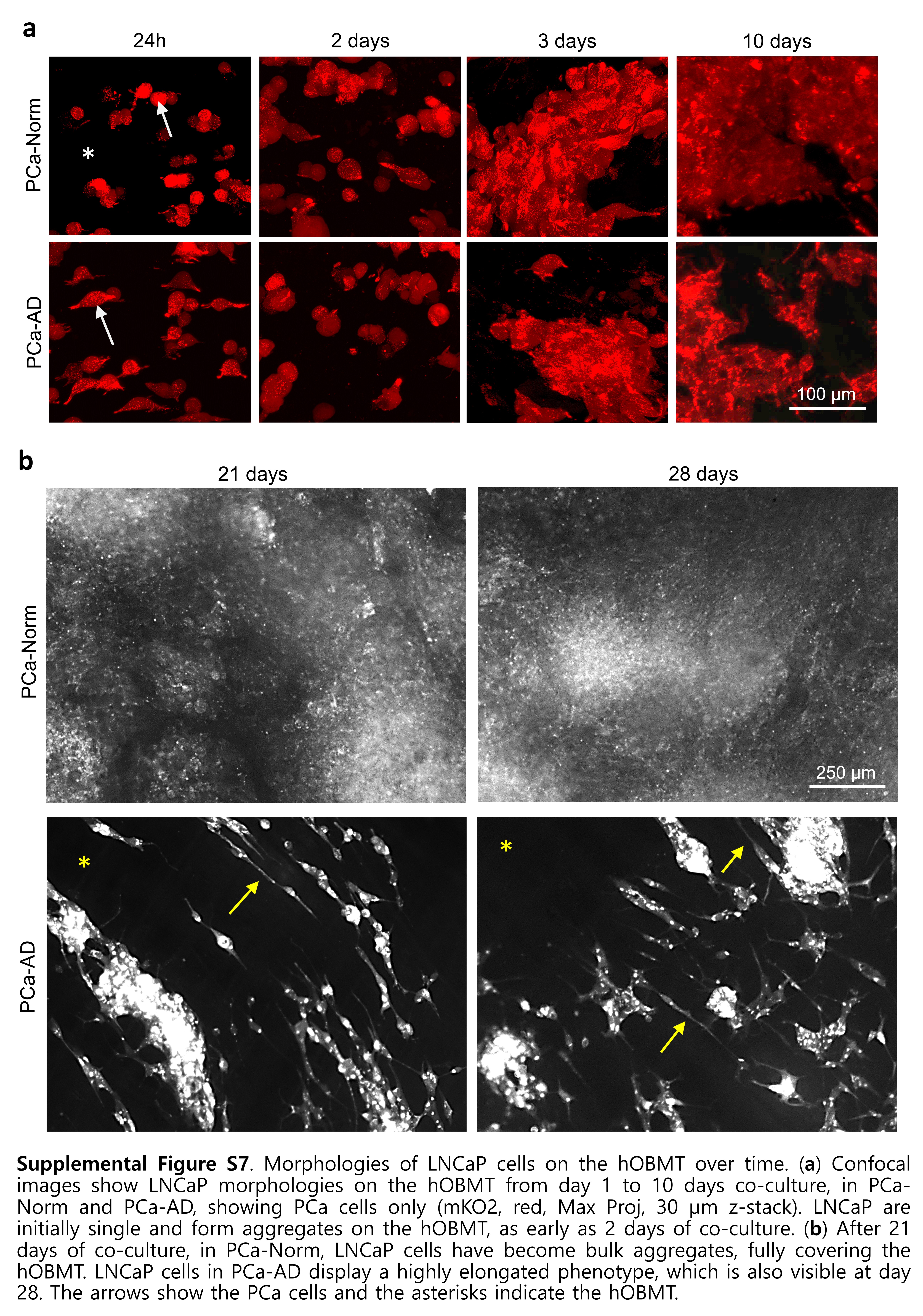

Supplement: Supplementary file 8 — Supplemental Figure S7 [file 41413_2019_49_MOESM8_ESM.jpg]

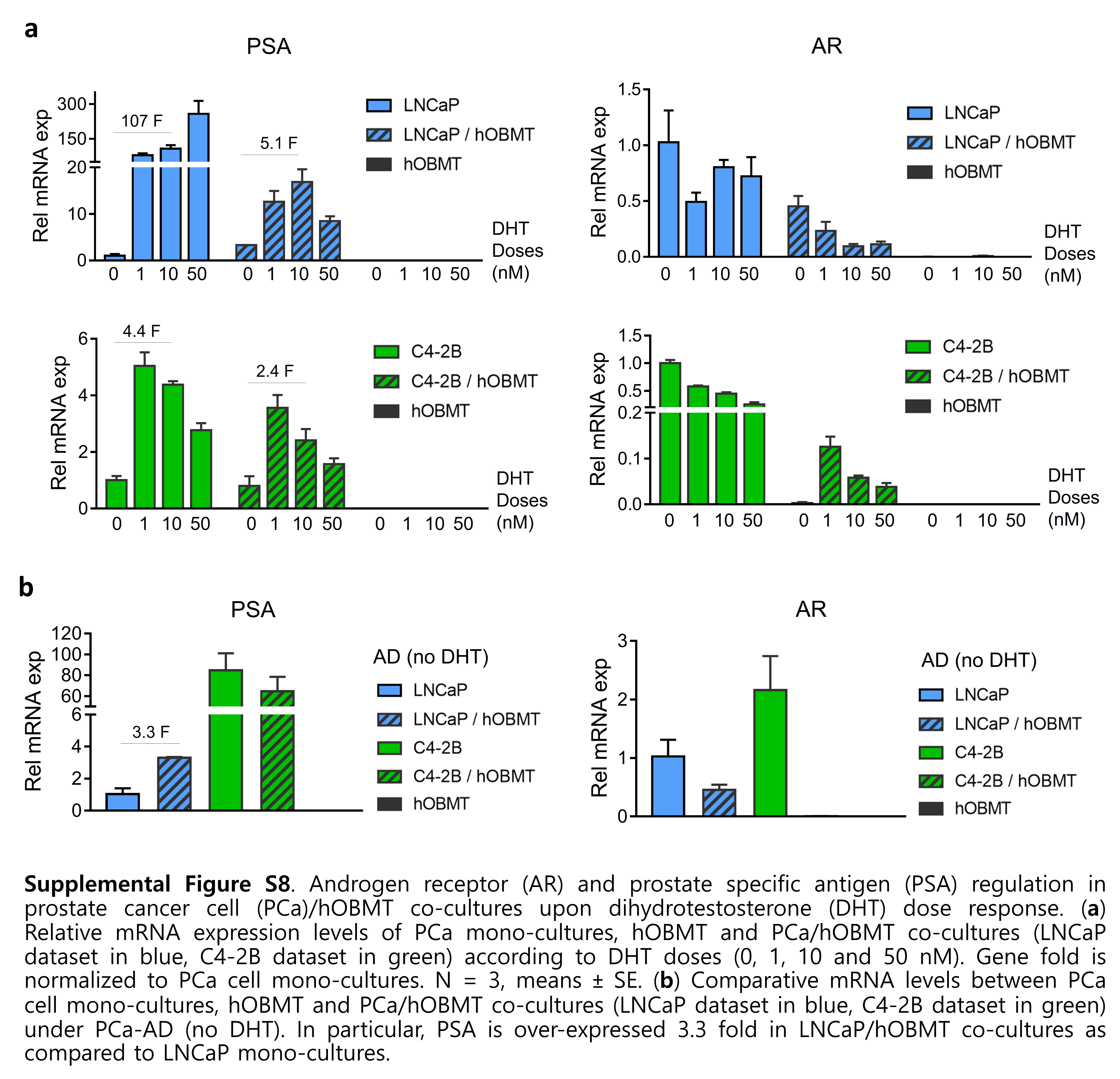

Supplement: Supplementary file 9 — Supplemental Figure S8 [file 41413_2019_49_MOESM9_ESM.jpg]

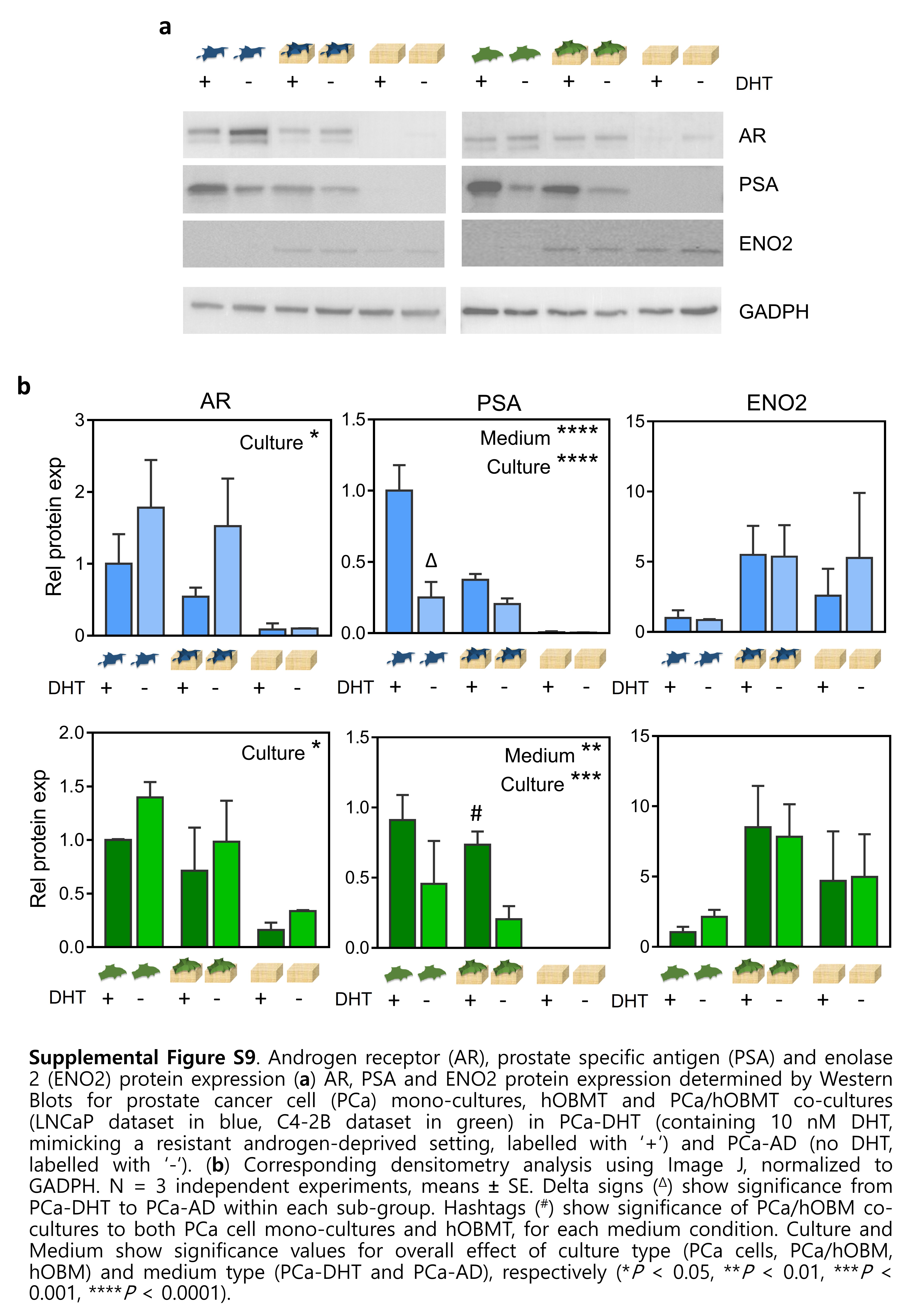

Supplement: Supplementary file 10 — Supplemental Figure S9 [file 41413_2019_49_MOESM10_ESM.jpg]

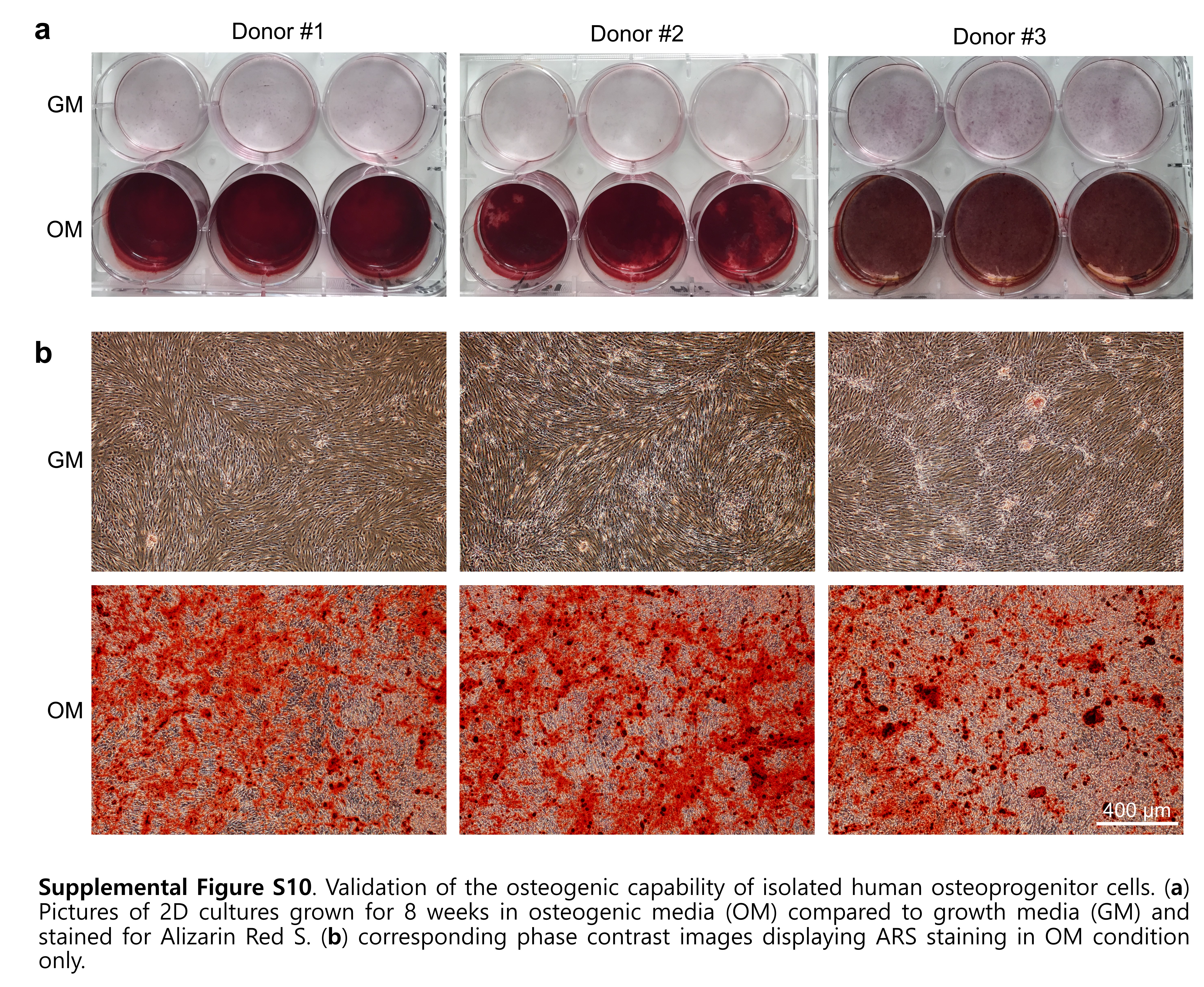

Supplement: Supplementary file 11 — Supplemental Figure S10 [file 41413_2019_49_MOESM11_ESM.jpg]
